# Mechanical, microstructural, and radiation shielding characteristics of sustainable high-strength concrete incorporating recycled wastes blended powders

**DOI:** 10.1038/s41598-025-23824-z

**Published:** 2025-11-18

**Authors:** Aya K. Elbauomy, Islam N. Fathy, Islam M. Nabil, Ahmed Elgabry, Gehan A. Hamdy, Mohamed S. Saif

**Affiliations:** 1 Department of Civil Engineering, Faculty of Engineering at Shoubra, Benha University, Cairo, Egypt; 2 Construction and Building Engineering Department, October High Institute for Engineering & Technology, Giza, Egypt; 3 Physics Department, Faculty of Science, Fayoum University, Fayoum, Egypt; 4 Housing and Building National Research Center (HBRC), Giza, Egypt

**Keywords:** Dealuminate metakaolin (DK), Limestone (LS), Silica fume (SF), Mechanical properties, Radiation shielding, High strength concrete (HSC), Engineering, Materials science

## Abstract

This study investigated the influence of blended powders of dealuminated metakaolin (DK), limestone (LS), and silica fume (SF) as partial cement replacements on the properties of high-strength concrete (HSC). Nine concrete mixes were designed, including a control mix and mixes incorporating binary, ternary, and quaternary blends of SF, LS, and DK at varying cement replacement levels. The experimental program evaluated the physical properties (slump, setting times, consistency), mechanical properties (compressive and tensile strengths), and microstructure (SEM, XRD, and EDX analysis) of the investigated concrete mixes. Furthermore, radiation shielding properties of the produced concretes were assessed using Monte Carlo (MC) simulations and Phy-X software. The analysis covered both γ-rays and fast neutrons. Results showed that quaternary blends of DK, LS, and SF reduced slump due to higher water demand, while their increased content enhanced compressive and tensile strengths. Optimal strength values were achieved with specific blend ratios: mix 4 (10% DK) for binary blends, mix 6 (15% SF+10% DK) for ternary blends, and mix 9 (15% SF+10% LS+10% DK) for quaternary blends. These optimal mixes exhibited compressive strength increases of 37.3%, 43.35%, and 23.4%, and tensile strength increases of 15.3%, 32.4%, and 22.25%, respectively, compared to the control mix. SEM analysis showed fewer voids and microcracks and a denser microstructure in the optimal replacement mixes. Furthermore, XRD and EDX analyses confirmed that DK, LS, and SF promoted the formation of calcium silicate hydrate (CSH) and calcium aluminate hydrate (CAH) through pozzolanic reactions. The γ-ray attenuation tests indicated modest improvement in γ-ray shielding capacity of HSC, with DK10 and SF15LS10DK10 mixes recording the highest linear attenuation coefficients (LAC). This improved performance is attributed to their elevated densities (2.47, 2.45 g cm^−3^) and substantial iron content (1.53% and 1.92%, respectively). Additionally, DK10, SF15LS10, and SF15LS10DK10 mixes exhibited excellent neutron shielding, achieving a removal cross-section (FCS) value of 0.086 cm⁻^1^, with the lowest half value layer (HVL_FCS_) of 8.059 cm, and relaxation length (λ_FCS_) of 11.627 cm.

## Introduction

Enhancing the performance and durability of concrete has driven significant research into the production of HSC^1^. The American Concrete Institute (ACI) defines high strength concrete (HSC) as concrete with a minimum compressive strength of 40 MPa^2^. Achieving this strength necessitates specific mix design parameters, including a low water-to-binder ratio (w/b) and a high binder content^3^. Additionally, supplementary cementitious materials (SCMs) comprising naturally or artificially derived aluminous and siliceous materials are commonly incorporated into ordinary Portland cement (OPC) to improve concrete properties. In the presence of moisture, SCMs react with cement hydration products, yielding additional strength through the formation of calcium silicate and calcium aluminate silicate hydrates^4^. Common SCMs include silica fume (SF), fly ash (FA), ground granulated blast-furnace slag (GGBS), and metakaolin (MK)^5,6^. Despite the numerous advantages of HSC such as its superior strength, durability, and cost-effectiveness relative to the strength achieved, its performance after exposure to elevated temperatures remains a concern^7^. Therefore, evaluating the elevated temperature resistance of HSC is crucial^8^.

The increasing focus on sustainability necessitates a reduction in ordinary Portland cement, making SCMs a crucial solution for cement replacement^9^. Portland cement, the primary binding material in concrete, has seen increased production due to population growth and the continuous increased demand in the construction sector. This surge has resulted in significant natural resource and energy consumption, and contributes of (7–8%) of the total CO_2_ emissions resulted from limestone calcination and fossil fuel combustion^10^. This coincides with industrialization, which has led to environmental pollution through greenhouse gas emissions and the accumulation of waste in landfills, impacting water, soil, and air quality^11^. Consequently, global efforts are focused on recycling by-products and waste materials in construction to enhance material performance, reduce costs, and address environmental concerns^12,13^. Various industrial by-products, such as SF^14^, FA^15^, blast-furnace slag^16^, granitic rock dusts^17^, and agricultural waste ashes like basil plant ash^18^, rice husk ash and sugarcane bagasse ash^19^, exhibit proved pozzolanic reactivity. Their chemical composition, characterized by siliceous or siliceous and aluminous materials, enables them to react with calcium hydroxide Ca(OH)_2_, thereby generating additional hydration products^20^. Consequently, the performance of blended cement depends strongly on the characteristics of the used industrial by-products. These characteristics primarily include their chemical composition, surface area, particle geometry, particle size distribution, and degree of amorphousness.

Generally, SCMs contribute to concrete performance through either pozzolanic or hydraulic reactions within the pore solution^21^. SF is one of the most commonly used materials in concrete mixtures, whether as an additive or as a partial replacement for cement. The low-content inclusion of SF in concrete mixes is particularly advantageous due to its significant reactivity with Ca(OH)_2_ formed during cement hydration process^22^. However, using high proportions of SF in concrete may negatively affect several properties such as workability, which tends to decrease due to the extremely fine particle size and high surface area value of SF particles. This fineness increases water demand and contributes to the densification of the concrete mix, making it more difficult to handle and place. Therefore, the use of SF in concrete mixtures is typically accompanied by the addition of superplasticizers, which help to enhance workability, improve dispersion of particles, and maintain the desired flow characteristics without increasing the water-to-cement (w/c) ratio^23^. In ultra-high-performance concrete (UHPC) mixes, the proper use of SF content, typically at addition or replacement levels ranging from 5 to 15%, helps reduce concrete porosity, bleeding, and permeability as a result of its extremely fine particle size and high pozzolanic reactivity^24^. These properties enable SF to fill the micro-voids between cement particles and react with Ca(OH)_2_ to form additional calcium silicate hydrate (C–S–H), which densifies the microstructure and improves the overall durability and impermeability of the concrete matrix.

Limestone powder (LS) is an ultra-fine byproduct which is generated during the cutting and processing of large limestone rocks and characterized by its exceedingly small particle size and high specific surface area. These properties render it a significant health hazard, particularly to the respiratory system upon inhalation, and contribute to notable environmental problems through its airborne dispersion^25^. Consequently, the environmentally sound management of this waste material, via its utilization as either an additive or a partial cement replacement in concrete mixtures, presents a sustainable solution. This approach not only ensures safe disposal but also offers the potential to enhance the performance characteristics of the concrete. Limestone particles can be used as partial replacement materials for either cement or fine aggregate, depending on their particle size. When used at appropriate replacement levels, they can lead to improved mechanical properties of the resulting concrete in both cases^26^. The presence of calcium carbonate (CaCO_3_), the main component of LS can accelerate cement hydration process. It especially affects tricalcium silicate activity and promotes more carbosilicate hydrate formation. These interactions collectively induce significant physico-chemical modifications within the system^27^. Moreover, the intrinsic filler effects of LS are credited with improving concrete density and strengthening the interfacial transition zone between the matrix and the aggregate, which are key factors in achieving superior concrete performance^28^. The small particle size of LS fillers intrinsically enhances packing density and reduces free water within concrete, facilitating a lower w/b ratio and increased lubricating paste volume. These benefits are critical for achieving essential flowability and segregation resistance for concrete^29^. Crucially, the synergistic use of LS alongside other pozzolanic materials or natural pozzolans provides a robust means to upgrade the overall performance of concrete^30^.

Due to the rapid expansion of industrialization and population, the accumulation of hazardous waste has reached critical levels^31^. Recycling these wastes in concrete production represents a sustainable approach. In addition to ensuring their safe disposal, they can also enhance various concrete properties when used as partial or complete replacements for either aggregates or cement^32–35^. In Egypt, where conventional bauxite resources are scarce, alum is manufactured from locally sourced kaolin and is extensively used, particularly in water treatment^36^. The production process involves sulfuric acid leaching of metakaolin, which leads to dealumination and the generation of an acidic, silica-rich byproduct known as dealuminated metakaolin (DK)^37^. The large-scale disposal of DK in landfills presents a significant environmental hazard, highlighting the urgent need for sustainable utilization methods. Consequently, investigating DK’s potential as a sustainable additive or partial replacement in cementitious systems has become a crucial area of research, aiming to mitigate environmental harm while simultaneously improving concrete performance. The profound pozzolanic efficacy of DK has been extensively documented, with some studies indicating its performance exceeds that of SF. Due to its enriched silica content, DK promotes the formation of silica-rich hydration products that refine the pore structure, enhance mechanical strength, and improve durability in concrete. In comparison, conventional metakaolin contains higher levels of reactive alumina alongside silica, which favors the generation of hydration phases, contributing to strength and durability but with a different balance of hydration products^38^. Thus, DK provides a denser microstructure and superior resistance in aggressive environments due to its silica-dominant composition. Mostafa et al. (2001) elucidated that the acid leaching treatment applied to DK results in a substantial increase in its specific surface area (up to 90.5 m^2^/g) and the generation of highly reactive amorphous silica, underpinning its superior pozzolanicity^39^. This heightened reactivity was corroborated by Abo-El-Enein et al. (2013), who observed that DK achieved lime fixation in a remarkably short 12-h period, a process that demanded 28 days for both MK and SF, thereby underscoring DK’s rapid pozzolanic kinetics^40^. Experimental studies, such as Abdelalim et al. (2008), explored the effects of substituting ordinary Portland cement (OPC) with varying percentages of DK. Their findings reported an increase in water of consistency (e.g., a 16% rise at 20 wt% DK) and a slight reduction in flowability (4% at 20 wt% DK). The study concluded that 10 wt% DK represented the optimal replacement level for enhancing mechanical properties^37^. Similarly, Moselhy (2018) reported modest improvements in compressive strength (2.7%, 2.1%, and 0.6% for 5, 10, and 15 wt% DK replacements, respectively)^41^.

From another side, the radiation shielding behavior of HSC was investigated in the present study. Radiation can be classified into ionizing or non-ionizing radiation regarding the radiation energy level^18^. Ionizing radiation refers to high-energy particles or electromagnetic waves that have sufficient energy to remove tightly bound electrons from atoms, thereby creating ions^42^. This process can cause significant biological damage, including cell mutation and tissue destruction, and poses serious risks to both human health and the environment if not adequately controlled. Therefore, studying the shielding effectiveness of concrete against ionizing radiation is crucial for ensuring the safe use of this material in environments exposed to such hazards. Understanding the attenuation performance is particularly important because it determines the ability of concrete not only to provide structural integrity but also to function as an effective barrier against hazardous radiation. This dual role becomes essential in nuclear facilities, medical centers, nuclear research centers, and other radiation-prone applications, where both mechanical strength and protective capacity must be simultaneously optimized. Heavyweight concrete remains the most widely used type of radiation shielding concretes due to its high density, ability to incorporate heavy aggregates such as barite, magnetite, or hematite, and its proven effectiveness in attenuating both gamma rays and neutrons^43^. However, recent research has increasingly focused on exploring alternative approaches to improve the shielding capacity of other types of concrete. Such approaches include the incorporation of SCMs, industrial by-products, and nanomaterials, as well as the partial replacement of conventional aggregates with high density aggregates^44,45^. These modifications often lead to the refinement of the microstructure, reduction of voids and cracks, and improvement of matrix–aggregate bonding, which together enhance the overall density and homogeneity of the concrete. In addition, the inclusion of materials with higher atomic numbers, such as barite, iron oxides, or lead-based compounds, increases the probability of photon interaction through mechanisms such as photoelectric absorption and Compton scattering, thereby improving the attenuation of gamma rays^46^. Similarly, the presence of hydrogen-rich phases and improved compaction within the matrix contribute to higher neutron absorption and scattering.

In the current study, an investigation into the impact that DK, SF and LS have on the performance of HSC was carried out. This was achieved by designing nine concrete mixes that represent the binary, ternary, and quaternary use of the three materials. The experimental investigation involved an exhaustive characterization of the concrete’s fresh state and its resultant mechanical properties, notably assessing slump, compressive strength, and tensile strength. To gain insights into the microstructural evolution and elemental distribution, X-ray Diffraction (XRD),Scanning Electron Microscopy (SEM) and Energy-Dispersive X-ray Spectroscopy (EDX) analysis were conducted. For the critical assessment of radiation shielding capabilities, specimens demonstrating optimum compressive strength were chosen for testing. These specimens were then assessed for radiation shielding efficiency, utilizing the Monte Carlo (MC) simulation code and Phy-X software, to quantify the most important radiation shielding parameters such as linear attenuation coefficient (LAC) and half-value layer (HVL). Additionally, the MC code facilitated the simulation of fast neutron removal cross-section, half-value length (HVL_FCS_), and relaxation length (λ_FCS_​). The results of the EDX test, along with the measured density values of the specimens, served as the primary input parameters for the radiation shielding behavior simulations of the HSC mixes designed in the present study, conducted using both MC method and the Phy-X software. It’s worth mentioning that the potential of limestone powder and de-aluminated kaolin to influence the attenuation performance of HSC against ionizing radiation has not been previously reported. Their combined role in modifying the microstructure of concrete, altering density, and potentially enhancing photon and neutron interaction probabilities remains unexplored in the existing body of literature.

## Materials and experimental study

### Materials properties

**Fig. 1:**
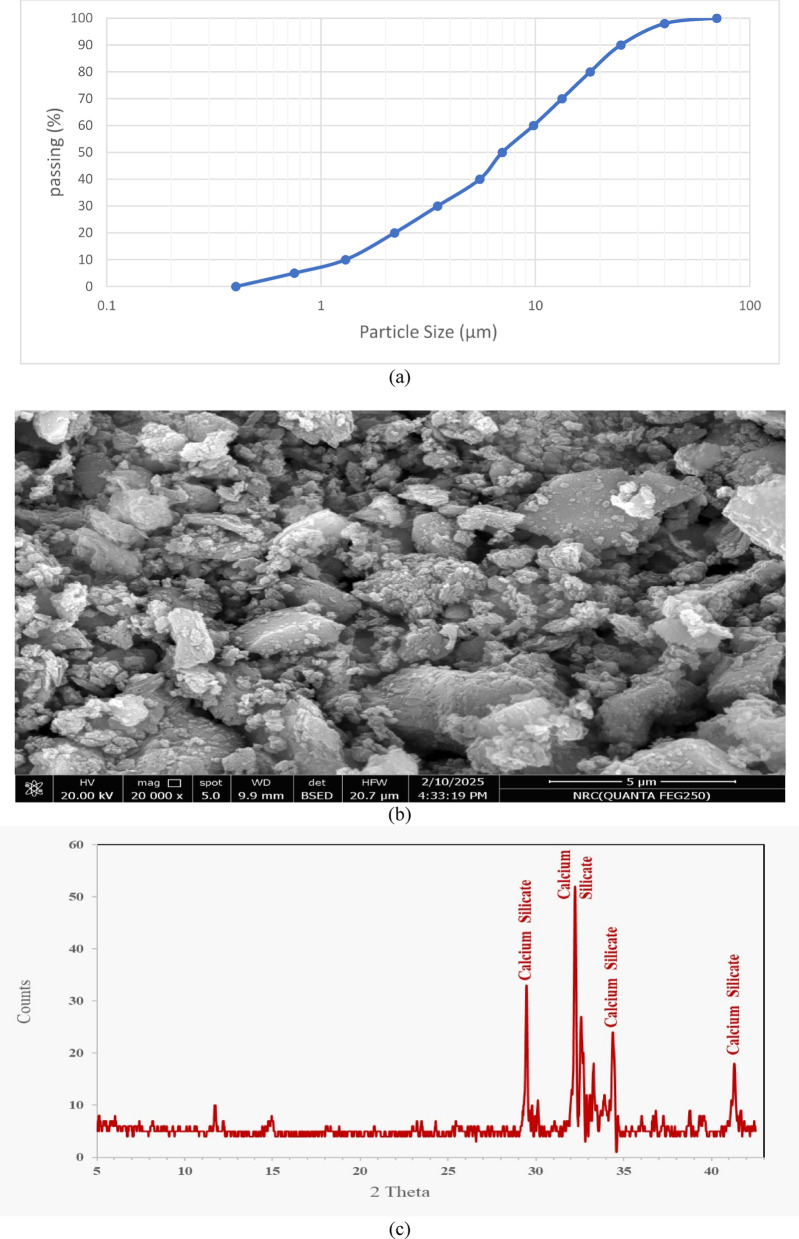
(**a**) Particle size distribution, (**b**) SEM image, and (**c**) XRD pattern of cement.

In this investigation, ordinary Portland cement (CEM I 52.5 N) which is available in the local market was used as the main cementitious binder material. This cement conformed to ASTM C 150 standards^47^, demonstrating a minimum 28-day compressive strength of 52.5 MPa. Its comprehensive physical, chemical, and mechanical properties are detailed in Table 1, while the particle size distribution, SEM image, and XRD pattern are introduced in Fig. 1. Particle size analysis shown in Fig. 1a indicated an average particle size of approximately 7 µm for the used cement.

**Table 1:** Physical, mechanical, and chemical characteristics of cement and waste blended powders.

Property	OPC	SF	LS	DK
Physical properties				
Color	Gray	Light Gray	White	Light yellow
Specific gravity	3.15	2.15	2.68	2.59
Surface area (cm^2^/g)	3500	25,000	5000	20,000
Average particle size	7 µm	1 µm	12 µm	5 µm
Initial setting time (min)	160	–	–	–
Final setting time (min)	280	–	–	–
Mechanical strength at various ages (MPa)				
3 days	26.2			
7 days	37.1			
28 days	53.4			
Chemical composition (% weight)				
SiO_2_	20	96.15	3.51	60.34
CaO	63.33	0.24	54	0.50
Al_2_O_3_	5	0.14	0.31	10.22
Fe_2_O_3_	3.36	1.15	0.17	0.80
MgO	1.9	0.14	0.80	0.06
SO_3_	2.6	0.12	0.055	12.66
Na_2_O	0.15	0.13	0.03	0.16
K_2_O	0.4	0.22	0.4	0.08
P_2_O_5_	–	–	–	0.02
MnO	–	–	–	0.01
Cl	–	–	–	0.01
TiO_2_	0.3	–	–	2.78
LOI*	3.85	2.13	4.3	12.01

*LOI, loss on ignition at 1000 °C

The current investigation utilized three distinct waste-derived powders: silica fume (SF), limestone powder (LS), and dealuminated kaolin (DK). For SF, it’s a byproduct of silicon and ferrosilicon alloy manufacturing and predominantly comprises amorphous silicon dioxide. The SF employed is sourced from the local Egyptian Ferro Alloys Corporation satisfied ASTM C1240-15 standards^48^ with amorphous SiO_2_ content of 96.15%, indicative of its high purity and pozzolanic potential. On the other side, the used LS powder, obtained from Egyptian limestone mills, is consists of fine and white CaCO_3_ granules, which are finer than cement with surface area of 5000 cm^2^/gm and typically functions as partial cement replacement material.

**Fig. 2:**
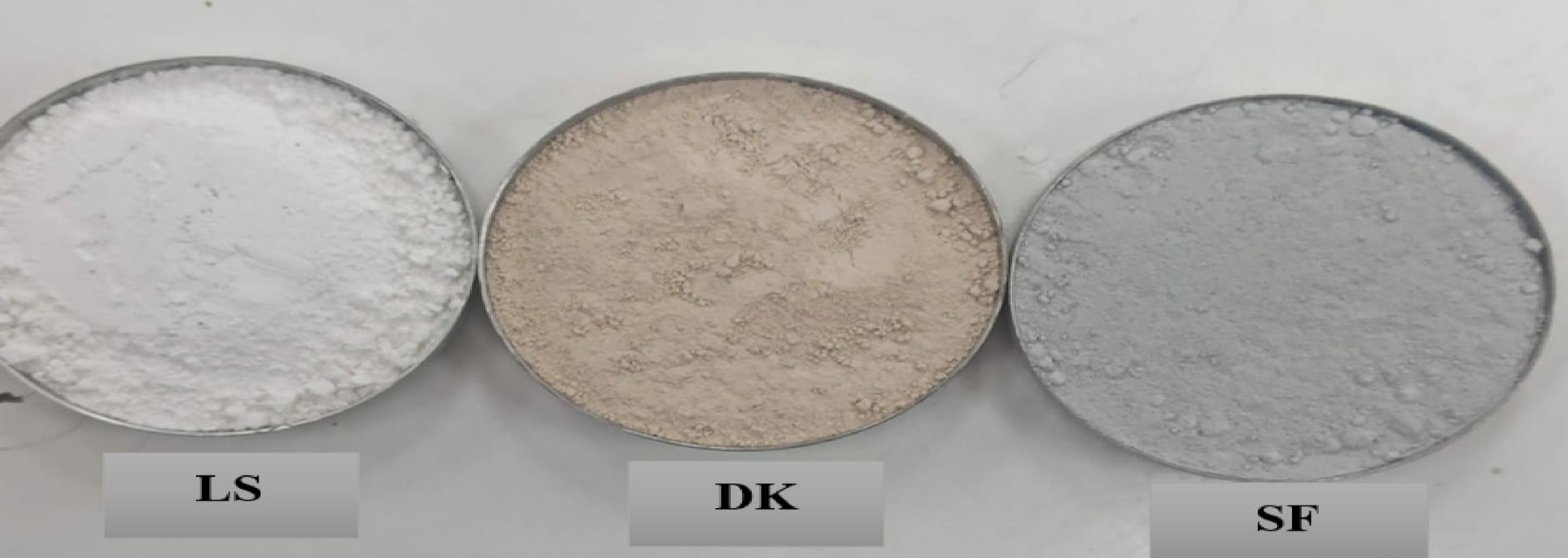
Appearance of the used wastes powder.

**Fig. 3:**
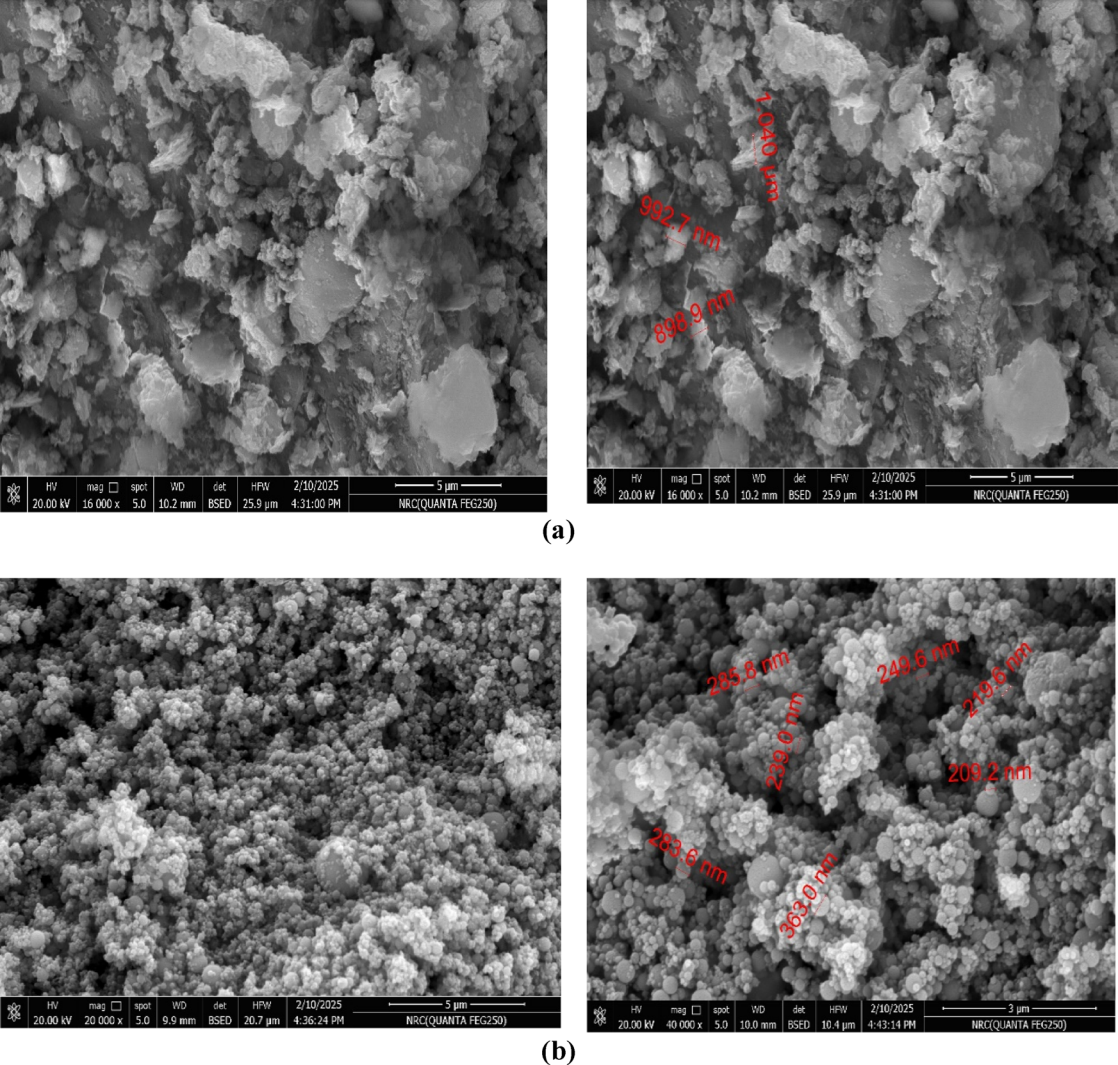
SEM images of (**a**) DK and (**b**) SF.

**Fig. 4:**
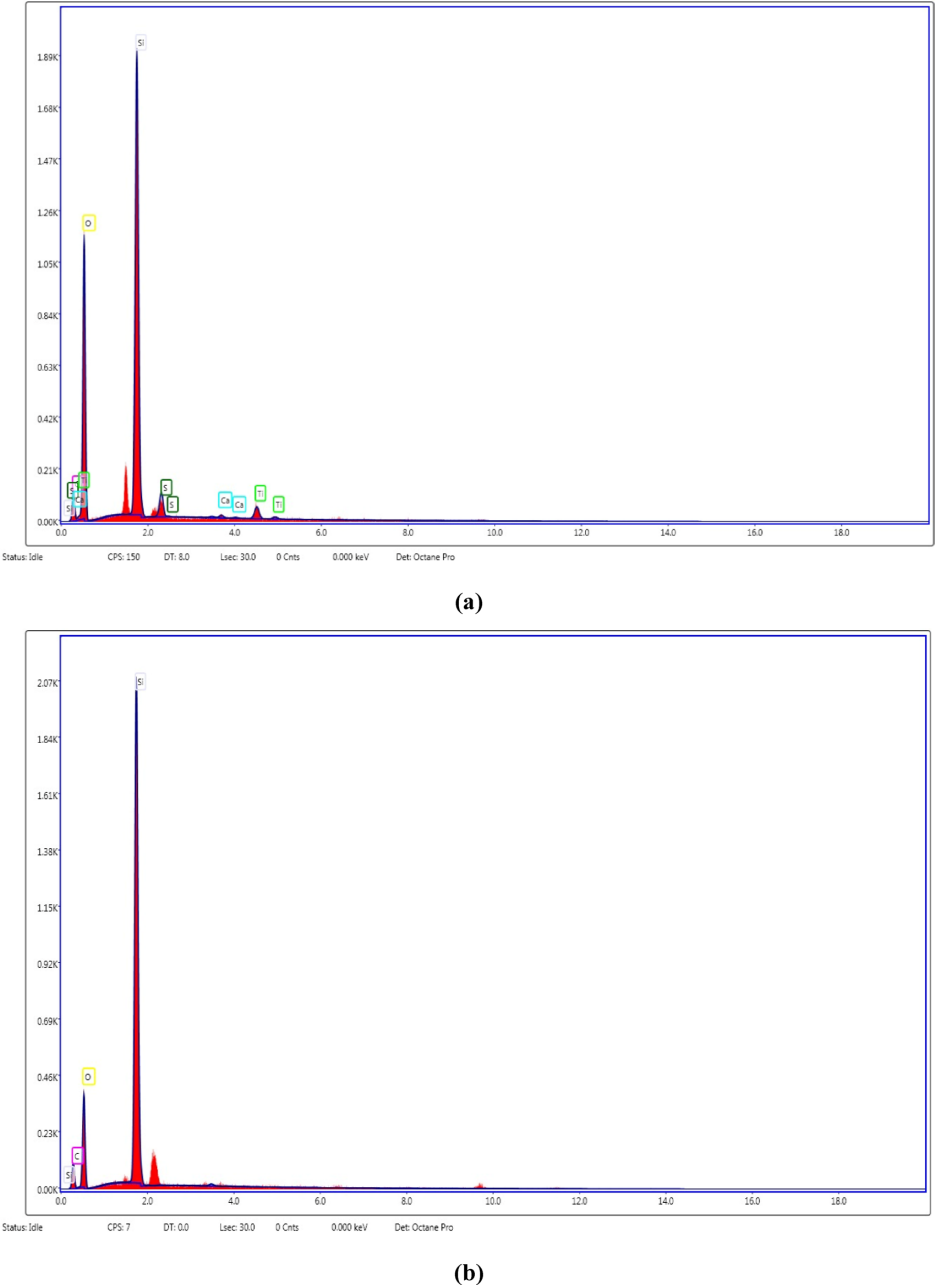
EDX patterns for (**a**) DK and (**b**) SF.

DK powder is a waste by-product generated by aluminium production industry, named dealuminated kaolin, is constantly increasing, and should be safely recycled for disposal. Safe disposal of DK is a crucial environmental issue. The used DK powder in this study was obtained from Egyptian Shaba Company in Abou Zaabl, Kalubia Egypt as shown in Fig. 2. Chemically, DK comprises over 70% silicate and aluminate compounds (Table 1), firmly classifying it as a pozzolanic material that reacts with Ca(OH)_2_ in the presence of water to generate additional hydration products. Thus, SF and DK are pozzolanic in nature, while LS serves as a filler. The microstructural analysis of DK and SF was investigated by SEM and EDX as presented in Figs. 3 and 4.

Regarding the aggregates used in the HSC mixtures in the present study, crushed dolomite with a maximum nominal size of 19 mm was used as coarse aggregate, while natural siliceous sand with a fineness modulus of 2.64 was employed as fine aggregate. Table 2 presents the results of sieve analysis for the particle size distribution of both coarse and fine aggregates, which were found to be within the allowable limits specified by ASTM C33-03 standards^49^. The physical properties of the aggregate types used in this study are presented in Table 3.

**Table 2:** Sieve analysis and grading limits of aggregates.

Sieve size (mm)	% Passing		Coarse aggregate specifications limits		Fine aggregate specifications limits	
	Coarse aggregate	Fine aggregate	Lower limit	Upper limit	Lower limit	Upper limit
25	100	100	100	100	100	100
19	100	100	90	100	100	100
9.5	36	100	20	55	100	100
4.75	7	100	0	10	95	100
2.36	1	98	0	5	80	100
1.18	0	70	0	0	50	85
0.6	0	45	0	0	25	60
0.3	0	24	0	0	5	30
0.15	0	3	0	0	0	10

**Table 3:** Physical properties of the used aggregates.

Aggregate type	Specific gravity	Bulk density (kg/m^3^)	Voids (%)	Absorption (%)	Crushing Value (%)	Fineness modulus
Crushed dolomite	2.66	1668	35	0.54	20	–
Sand	2.52	1728	32	0.85	–	2.64

**Fig. 5:**
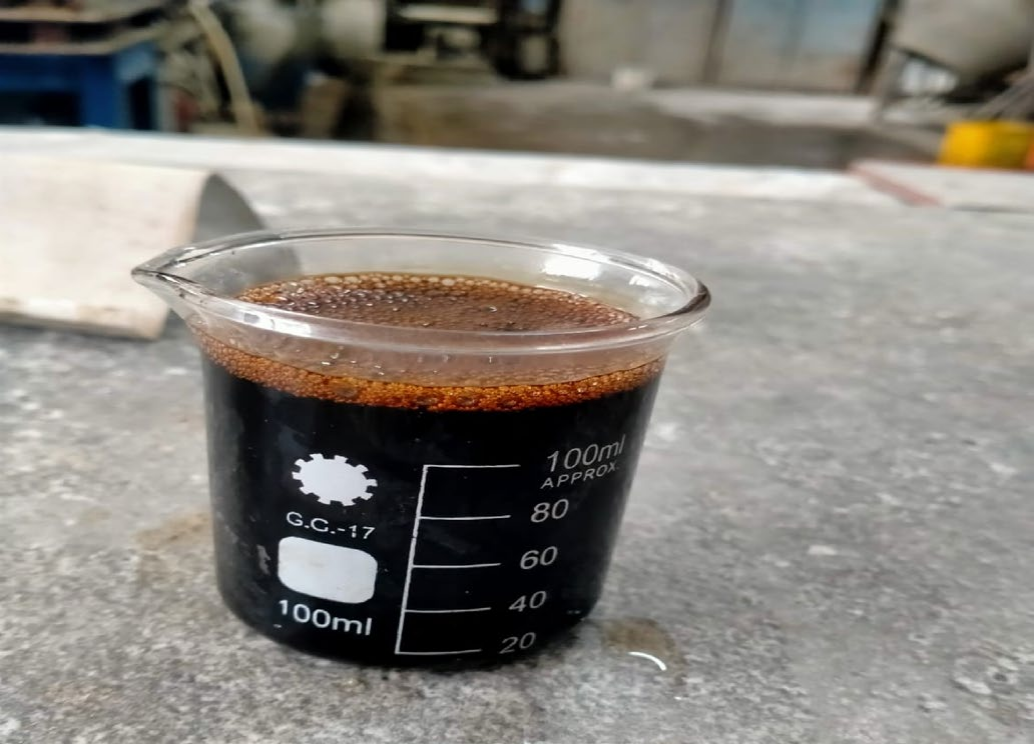
The used superplasticizer.

This study utilized ordinary drinking tap water for both concrete mixing and curing, ensuring full compliance with ASTM C1602/C1602M quality standards^50^. To effectively manage the increased water demand posed by the large surface area of the fine powders and to optimize workability, high range water reducer superplasticizer (SP) was incorporated into all designed HSC mixes, as depicted in Fig. 5. This chemical admixture, confirmed to meet ASTM C 494 Type G specifications^51^, possesses a brown liquid form with specific gravity of 1.20 kg/L, and a pH of 8. It was consistently dosed at 2% by weight of the cementitious materials. The fundamental role of this SP was to facilitate improved concrete flow without requiring additional water, thus allowing for reduced w/c ratio at an equivalent cement content.

### Mix design

To ascertain the influence of SF, LS, and DK as cement substitutes on HSC properties, a series of nine concrete mixes were formulated following the approach of Bentz et al.^52^, which emphasizes the design of ternary blends to optimize both performance and sustainability. The selected proportions of SF, LS, and DK were determined to achieve an optimal balance between mechanical performance and workability. Silica fume was incorporated at 15% replacement due to its high pozzolanic reactivity and its role in refining the pore structure, leading to significant strength enhancement. Limestone powder was introduced at 10% replacement, primarily to act as a filler and nucleation agent, improving particle packing and early hydration kinetics. Diatomite was employed at moderate replacement levels (5–10%) to exploit its high surface area and potential for improving durability-related properties. The binary, ternary, and quaternary blends were therefore structured to progressively examine the synergistic effects of these supplementary materials, and their proportions were optimized to align with both mechanical performance requirements and the targeted radiation shielding efficiency. For the main components of the concrete mix, each one cubic meter of the concrete mix was characterized by a baseline composition of 700 kg of cement, 975 kg of crushed dolomite, 525 kg of sand, and 210 kg of water, yielding an approximate w/c ratio of 0.3. Furthermore, superplasticizer was consistently incorporated at fixed ratio (2% by cementitious material weight) to ensure suitable workability. The complete specifications for these nine distinct mix designs are detailed in Table 4.

**Table 4:** Mix design of concrete mixes (kg/m^3^).

Mix	Type	Code	Cement Kg	SF Kg	LS Kg	DK Kg	Fine aggregate Kg	Coarse aggregate Kg	SP Kg	Water Kg
1	Control	CO	700	–	–	–	525	975	14	210
2	Binary	SF15	595	105	–	–	525	975	14	210
3	Binary	DK5	665	–	–	35	525	975	14	210
4	Binary	DK10	630	–	–	70	525	975	14	210
5	Ternary	SF15DK5	560	105	–	35	525	975	14	210
6	Ternary	SF15DK10	525	105	–	70	525	975	14	210
7	Ternary	SF15LS10	525	105	70	–	525	975	14	210
8	Quaternary	SF15LS10DK5	490	105	70	35	525	975	14	210
9	Quaternary	SF15LS10DK10	455	105	70	70	525	975	14	210

### Mixing, casting and curing of concrete specimens

The present study employed distinct specimen geometries to quantify the impact of SF, LS, and DK as cement replacement materials on the compressive and tensile strength of concrete. Cubic molds (100 × 100 × 100 mm) facilitated compressive strength analysis, while cylindrical specimens (100 mm diameter, 200 mm height) were cast for tensile strength determination. Mix preparation involved the initial dry blending of concrete constituents, followed by the 2-mins incorporation of designated cementitious powders (cement/SF/LS/DK). During a subsequent five-minute mixing phase, 80% of the calculated mixing water and the full superplasticizer quantity were incrementally introduced. The remaining 20% of the mixing water was then added gradually over 2 mins, with continued mixing. Post-casting, specimens underwent compaction with an electric vibrator and surface leveling. Curing commenced 24 hours after casting, with specimens demolded and immersed in fresh, clean water at 25 ± 2 °C and > 95% relative humidity, until the 28-day testing regimen.

### Testing procedure

#### Fresh, mechanical, and microstructural properties

**Fig. 6:**
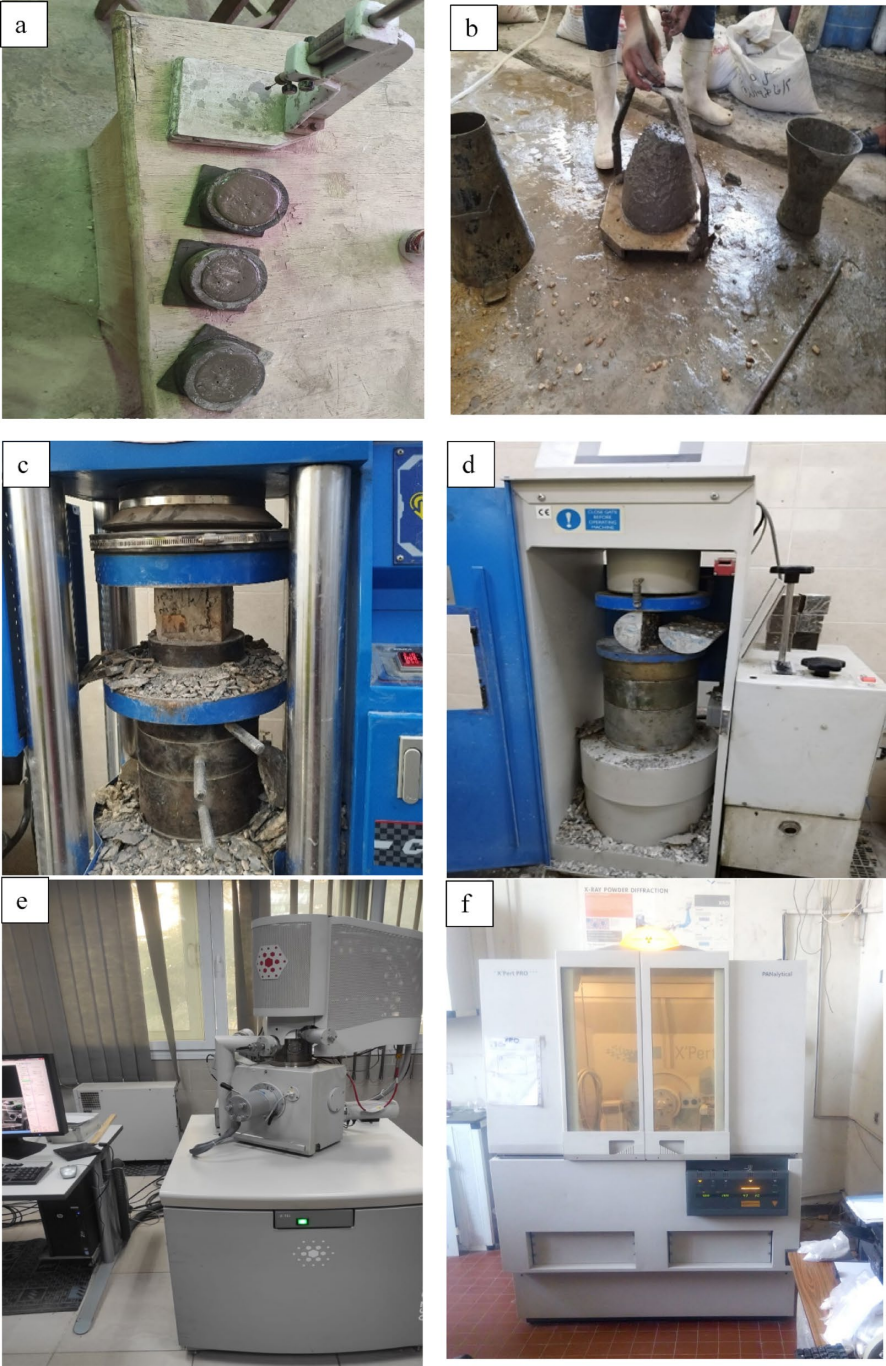
Testing producer for (**a**) Vicat needle, (**b**) Slump cone test, (c) Compressive strength machine, (d) Tensile strength machine, (e) SEM &EDX analysis apparatus, and (f) XRD analysis apparatus.

This study employed a multi-faceted approach to characterize the concrete’s fresh, mechanical, and microstructural attributes. Workability was quantified using the ASTM C143 standard slump cone test^52^. For mechanical property evaluation at 28 days of curing age, a 3000 kN uniaxial loading instrument available at concrete and materials lab at the Housing and Building National Research Center (HBRC) was utilized. As mentioned earlier, compressive strength was ascertained from 100 × 100x100 mm cubic samples, with the average of three replicates reported for each mix. Furthermore, splitting tensile strength was evaluated on 100 mm diameter by 200 mm height cylindrical specimens. For microstructural insights, XRD, SEM, and EDX analyses were performed. XRD analysis were executed on cement paste samples representing five of the investigated mixes and prepared by crushing and sieving the samples less than 75 µm (using diffractometer operating at 40 kV and 30 mA with a copper anode, available at HBRC). XRD analysis was employed to identify crystalline phases and evaluate the formation and consumption of hydration products, providing insights into the pozzolanic activity and microstructural development of the investigated mixes. For SEM analysis, it was conducted to observe the morphology and surface texture of the hardened concrete and paste samples, allowing visualization of the microstructural features such as the distribution of hydration products, and the presence of pores or microcracks that could influence the mechanical and durability performance of the mixes. For EDX analysis, it served as an effective method for quantitatively determining the elemental composition of concrete samples, enabling a comparative analysis of element percentages and identifying chemical shifts induced by the waste powders integration into HSC mixes. Figure 6 shows some of the machines and tools used in the experimental tests.

#### Radiation shielding measurements.

To assess the radiation shielding capabilities of the prepared concrete mixes, both simulation and computational approaches were employed. Monte Carlo simulations (MC) and Phy-X software were used to evaluate the radiation shielding performance against gamma and fast neutrons.

##### MC simulation

**Fig. 7:**
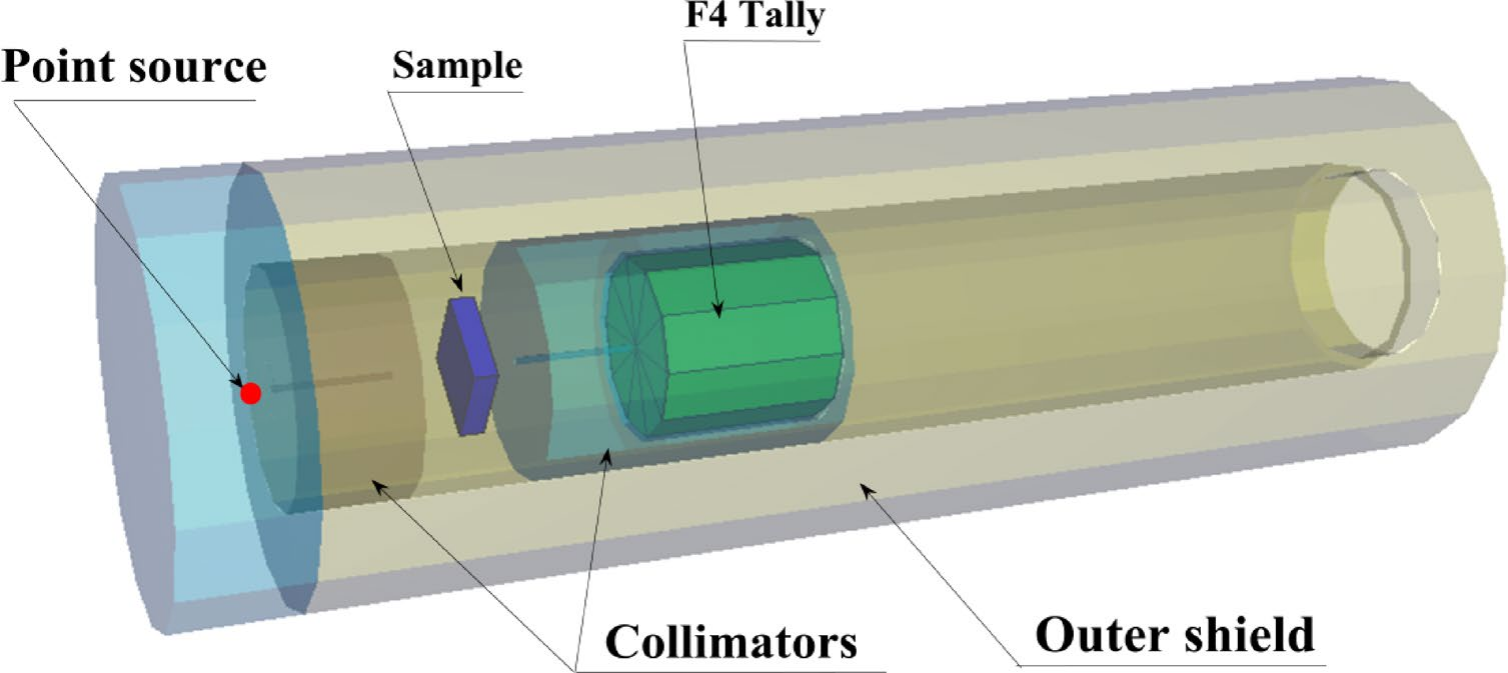
3-D view of the radiation attenuation simulation system used for the prepared HSC samples.

In this work, MC method was used to simulate the movement of natural particles as well as to predict the gamma/neutron radiation performance, in the energy range of gamma rays (γ_p_) at 0.015 ≤ γ_p_ ≤ 15 MeV. The target is to determine and compare the intensity of γ-rays before and after passing through the HSC specimens under study. This code is used in the study of radiation shielding, as well as calculating radiation doses in addition to detector design. The simulation code features operation across a wide range of energy levels, accommodating all different engineering designs and enabling rapid calculations. Several data lines must be available to create the input file for the code, which include the distance between the source and the detector, the dimensions of the source, the elemental composition, and the chemical composition of the samples under study. As shown in Fig. 7, the geometric configuration for the simulation was created using a pre-set 3D setup. The data about the used code was detailed in the previous published work^53^. Also, the Phy-X/PSD (PhyX) was used to validate the MC results. It is an online tool for figuring out radiation doses and shielding characteristics^54–56^.

The concrete shield consists of concrete cubes representing the control mix and the mixes containing various proportions of waste blended powders (SK, LS, and DK), each with dimensions of 100 × 100 × 100 mm, where the shield thickness is 10 cm. This thickness is substituted into the Beer-Lambert equation as follows^57^:

1 $${\text{LAC}},{\text{ cm}}^{{ - {1}}} = \left( {{1}/{\text{T}}} \right){\text{ ln }}\left( {{\text{C}}/{\text{C}}_{0} } \right)$$

where LAC is the linear attenuation coefficient (cm⁻^1^), T is the material thickness (cm), and C₀ and C are the initial and final counts of gamma-ray photons measured before and after shielding. Additionally, the values of other γ-ray attenuation parameters, such as the half-value thickness (HVT), tenth-value thickness (TVT), mean free path (MFP), effective atomic number (Z_ef_), transfer factor (TF) and radiation protection (RPE) were calculated using the following equations^58,59^.

2 $${\text{HVT}},{\text{ cm}} = {\text{ln }}\left( {2} \right)/{\text{LAC}}$$

3 $${\text{TVT}},{\text{ cm}} = {\text{ln }}\left( {{1}0} \right)/{\text{LAC}}$$

4 $${\text{MFP}},{\text{ cm}} = {1}/{\text{LAC}}$$

Each of these attenuation parameters provides unique insights into the shielding performance of concrete against γ-rays. The mass attenuation coefficient represents the attenuation per unit mass and is crucial for comparing materials with different densities, making it a fundamental parameter in material selection for radiation shielding. The HVT and TVT are the thickness of material required to reduce the gamma-ray intensity by half and tenth, offering a practical measure of the material’s efficiency in attenuating radiation. While HVT is often used for quick evaluations, TVT is more critical for scenarios requiring higher attenuation, highlighting the complementary nature of these parameters in assessing and optimizing shielding performance.

The MC method was employed in this study due to its robustness and widespread acceptance in radiation shielding research. This method operates by simulating the random paths of individual photons or neutrons as they interact with matter, thereby modeling the fundamental processes of scattering, absorption, and transmission. Each particle history is tracked through successive probabilistic events that are governed by well-established cross-section data, allowing the cumulative outcomes to provide statistically accurate predictions of radiation transport. By repeating a large number of such histories, the method yields reliable estimates of key shielding parameters such as LAC, transmission factor, and energy absorption. This particle-by-particle approach makes the MC technique particularly suitable for heterogeneous materials like concrete, where the complex composition and distribution of aggregates influence radiation shielding behavior. The accuracy of the MC technique has been validated in several previous investigations, where simulation results were found to be in strong agreement with experimental data^60–63^.

##### PhyX software

As an invaluable online resource, Phy-X facilitates the determination of critical radiation shielding parameters and dose assessments. This versatile tool quantifies essential radiation shielding coefficients such as LAC, MAC, MFP, HVT, TVT, and effective atomic number^64,65^. These computational capabilities are indispensable for the meticulous design of materials crucial to radiation protection, particularly against X-rays and gamma (γ)-rays. Users can readily input a substance’s chemical composition directly into the platform, enabling swift and comprehensive calculations across a broad spectrum of energies and diverse radiation sources. In the present study, the results of the EDX test for the elemental composition of the various HSC samples were used as input parameters in the Phy-X software to analyze the shielding efficiency of these samples against radiation.

## Results and discussion

### Fresh and physical properties

#### Slump test results

**Fig. 8:**
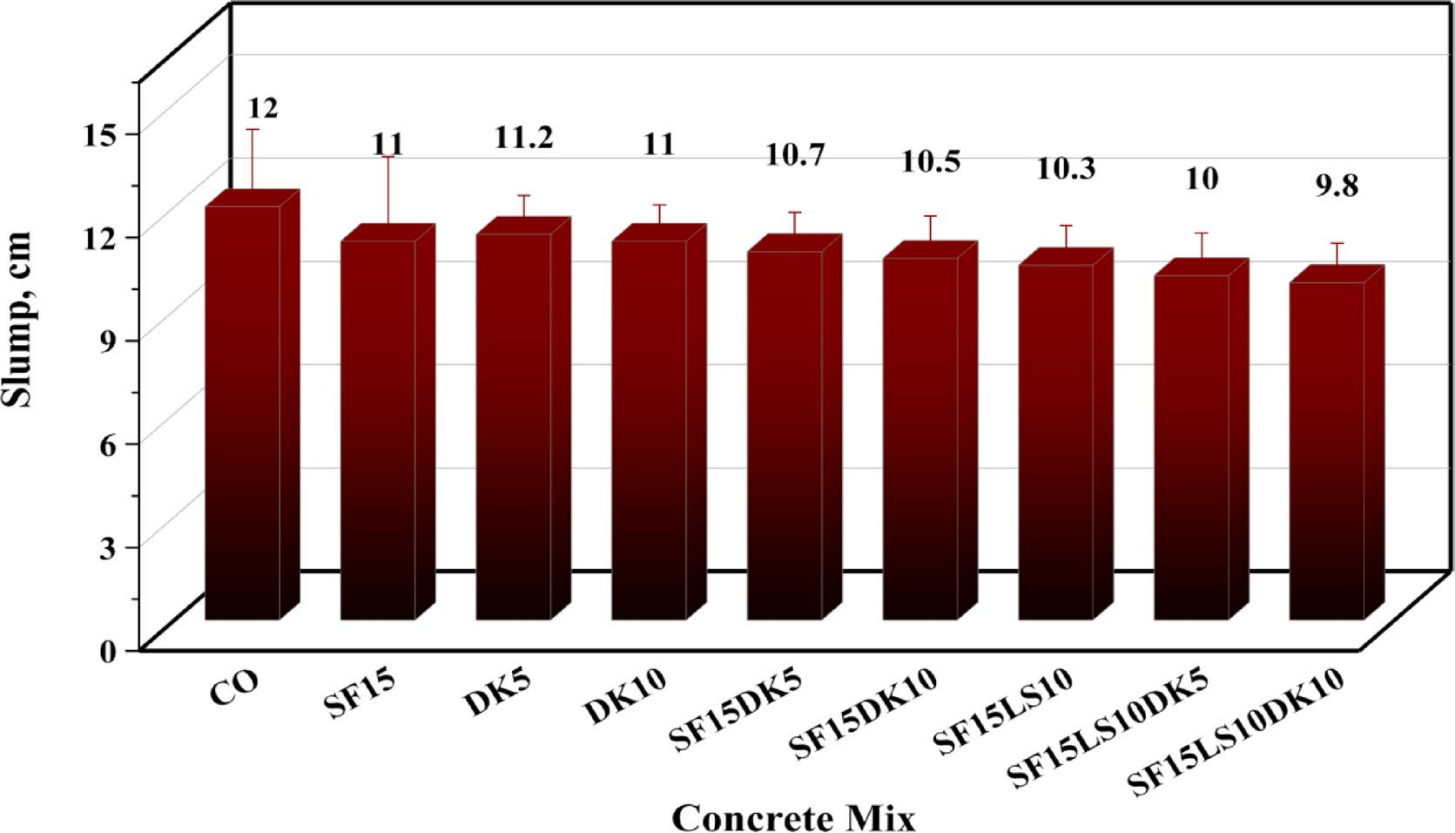
Slump values for various designed concrete mixes.

The incorporation of elevated replacement ratios of DK, SF, and LS demonstrably reduced the workability of the concrete mixture. This reduction is primarily attributable to the intrinsic higher fineness of these materials, which inherently increased the water demand of the mix due to the expanded surface area requiring greater hydration. Concomitantly, the fine and frequently angular, morphology of DK, SF, and LS particles augmented the internal friction within the concrete matrix, impeding its flow and consolidation. This collective effect manifested as a reduction in slump values, as illustrated in Fig. 8. The observed decrease in slump with increasing surface area of blended mixtures aligns with established principles; the replacement of cement with finely powdered materials generally necessitates higher water content to attain requisite workability^66^. This effect is more pronounced in concrete with pozzolanic wastes, where higher internal friction further reduces workability. The employment of superplasticizers or chemical admixtures is thus common to mitigate these effects. While pozzolanic waste materials can exacerbate this due to increased internal friction, the relatively modest slump decrease observed with LS powder in this study likely stems from the inclusion of a small percentage of superplasticizer (2% of cement weight). The observed decrease in slump values was marginally more pronounced when DK, SF, and LS were incorporated as a blend, in contrast to the sole inclusion of DK. Quantitatively, a 10% replacement of cement with DK yielded an 8.33% slump reduction. This increased to 14.16% for the 25% replacement encompassing 10% DK and 15% SF. The highest reduction, 18.33%, was recorded for the 35% replacement rate combining 10% DK, 15% SF, and 10% LS, representing individual, binary, and ternary blend cases, respectively. The most substantial workability reductions were observed in the ternary mixes Sf15Ls10dk10 and Sf15Ls10dk5, with slump decreases of 18.33% and 16.66%, respectively, relative to the control mix.

### Mechanical properties

#### Compressive strength

**Fig. 9:**
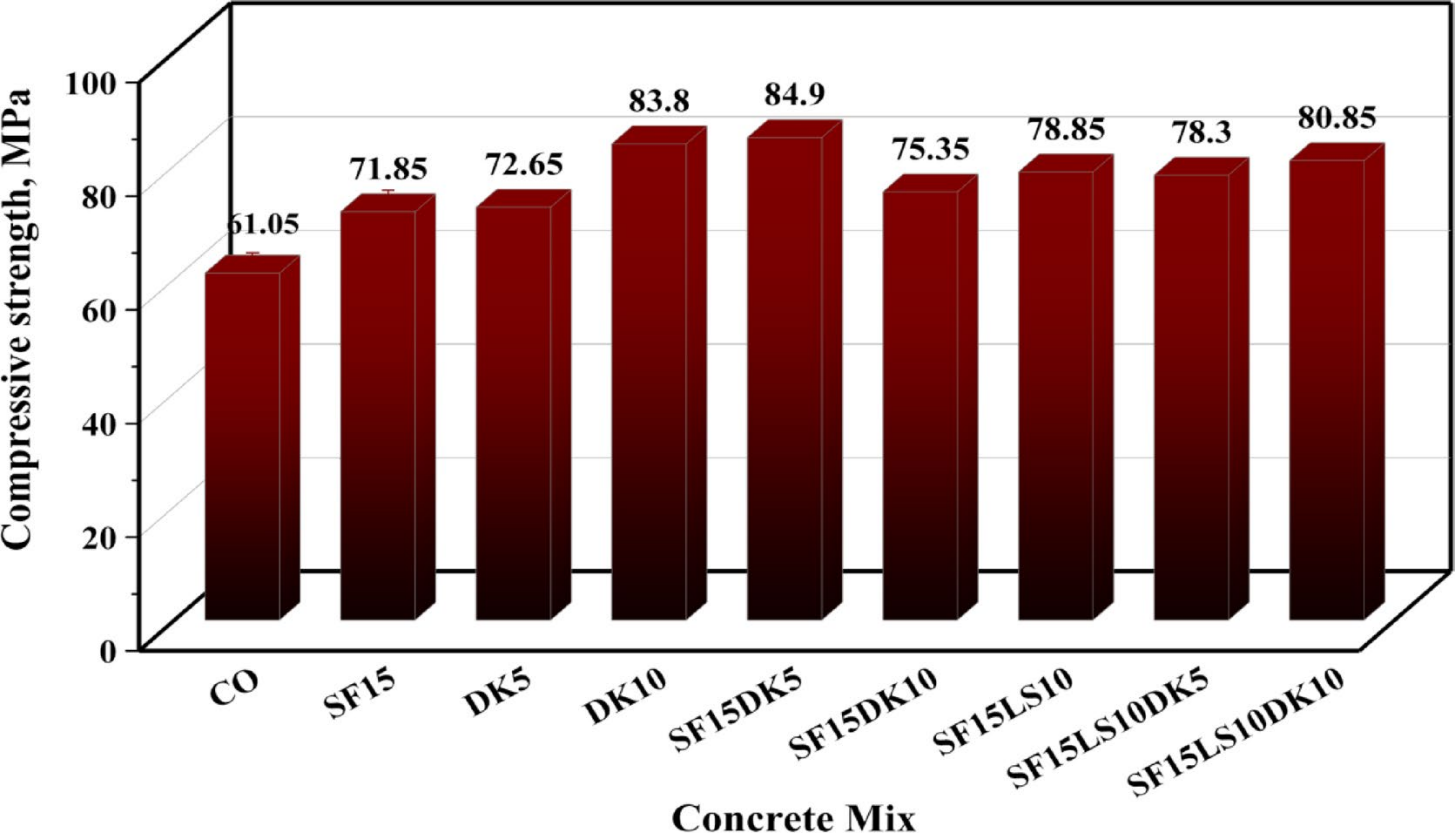
Compressive strength of all designed HSC mixes.

Figure 9 presents the effects of using SF, DK and LS on strength development of concrete at 28 days. The reference mix exhibited a compressive strength of 61.05 MPa. The addition of SF enhances significantly the compressive strength. 15% SF increases compressive strength by 17.7% compared to control OPC mix. The enhancement of the compressive strength of concrete can be attributed to the fact that SF particles can fill in the pores and cracks, densifying the microstructure and reducing the porosity of concrete. SF acts as seeding particles and can accelerate the precipitation of C–S–H on both the surfaces of SF and cement particles at early ages which is reflected in the remarkable improvement strength at the early ages as shown in the results.

DK incorporation increases compressive strength, and the percentage of improvement increases with increasing its content compared with OPC control mix. The compressive strength of HSC containing 5 and 10% DK increased by 19 and 37.2% respectively. This significant improvement in compressive strength may be attributed to the dual physical and chemical roles of DK dust. The physical role lies in the filling effect as a result of using high fineness DK dust which can densify the microstructure and fill the pores. The chemical role lies in the fact that DK is a pozzolanic material contains more than 70% amorphous silicate and aluminate in its chemical composition. Regarding binary blended cement replacement material in HSC, DK significantly improves compressive strength than SF at 28 days.

Regarding the ternary blend incorporating 15%SF with either 5%DK or 10%DK or 10%LS, the compressive strength increased by 39.1, 23.4 and 29.15%, respectively, compared to the control mix. The improved compressive strength for the mix containing (15%SF + 10LS) could be attributed to the important role of the filling effect of limestone particles as a result of using high Ls powder content which can densify the microstructure and fill the concrete pores. Previous studies showed that the compressive strength of concrete containing more than LS 10% decreased with increasing the content of cement replacement. It is clear from LS powder characterization that LS does not possess any pozzolanic activity, so it acts as a filler and inert material rather than a pozzolanic. The quaternary blended incorporating both (15%SF + 10%LS) with either 5% DK or 10%DK, increase compressive strength by 28.3 and 32.4% respectively higher than control mix.

#### Tensile strength

**Fig. 10:**
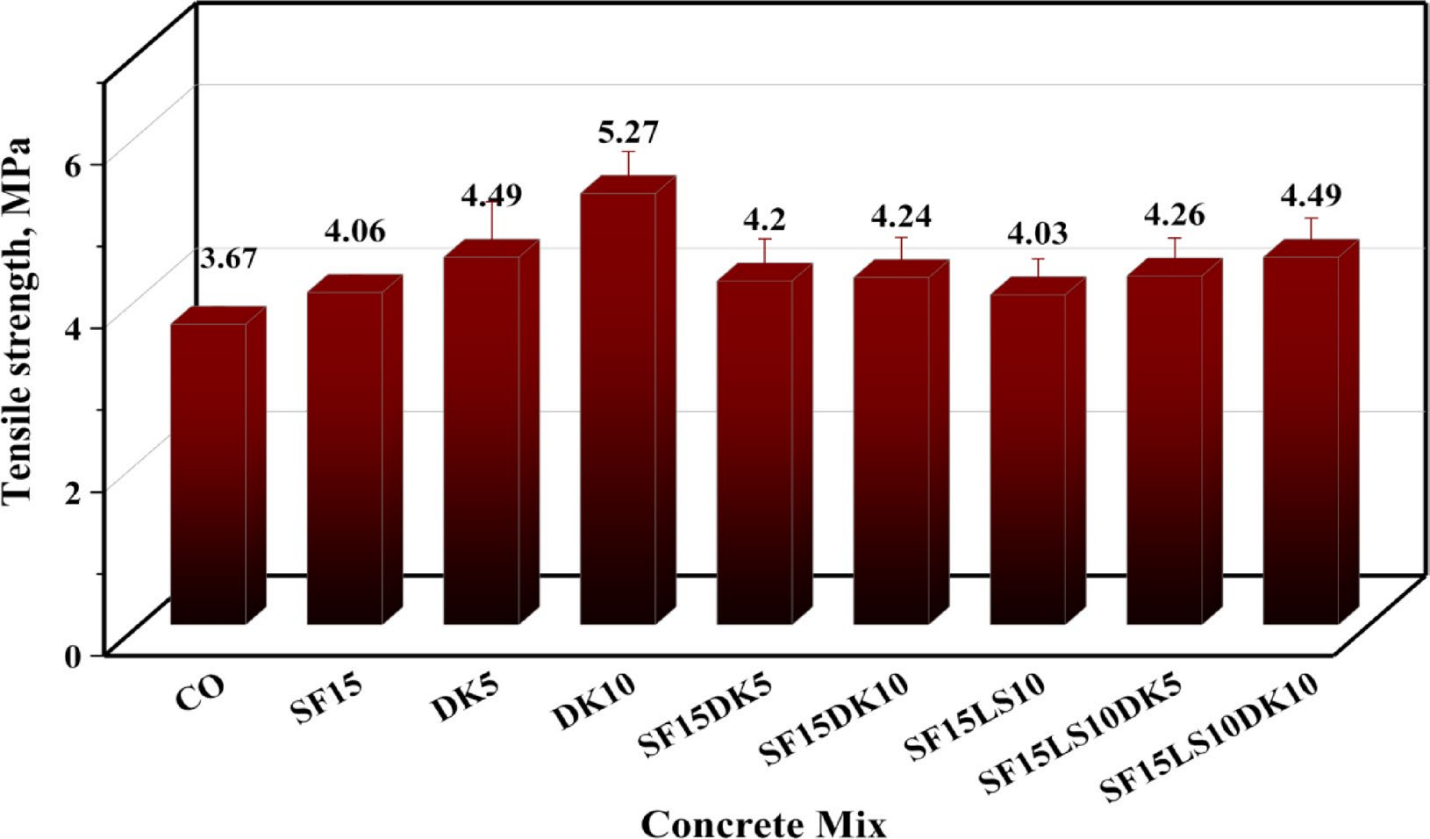
Tensile strength of all designed HSC mixes.

The influence of SF, DK, and LS on the 28-day splitting tensile strength of concrete is depicted in Fig. 10. A strong correlation was observed between these splitting tensile strength values and the previously obtained compressive strength results. In general, mixtures that exhibited higher compressive strength also demonstrated enhanced tensile strength, confirming the consistency of the mechanical performance trends. Concrete specimens containing 15% SF demonstrated an increase in tensile strength from 3.67 MPa for the control mix to approximately 4.06 MPa, signifying a 10.6% enhancement. Furthermore, concrete incorporating DK displayed a significant improvement in tensile strength, particularly as its value increased by 10% compared to the control mix. The results showed that 10% DK replacement ratio is the optimal, leading to an increase in tensile strength from 3.67 MPa to about 5.27 MPa, which constitutes a remarkable 43.35% increase. Similarly, the addition of 10% LS resulted in a substantial improvement in splitting tensile strength, with values rising from 3.67 MPa for the control mix to approximately 4.03 MPa, representing a 9.8% strength gain. The best performance in tensile strength for the concrete cylinders was recorded for the samples in mix 4, 6 and 9 which included DK, SF and LS powders. As previously noted, both materials act as SCMs with pozzolanic activity, contributing to the activation of cement hydration reactions and enhancing the mechanical strength of the concrete, especially at optimal replacement ratios. These optimal ratios were recorded at binary blended, ternary blended and quaternary blended mixes 4, 6 and 9 with improvements of 43.35%, 15.5% and 22.3% compared to the control mix, respectively.

### Microstructural properties

#### XRD results

**Fig. 11:**
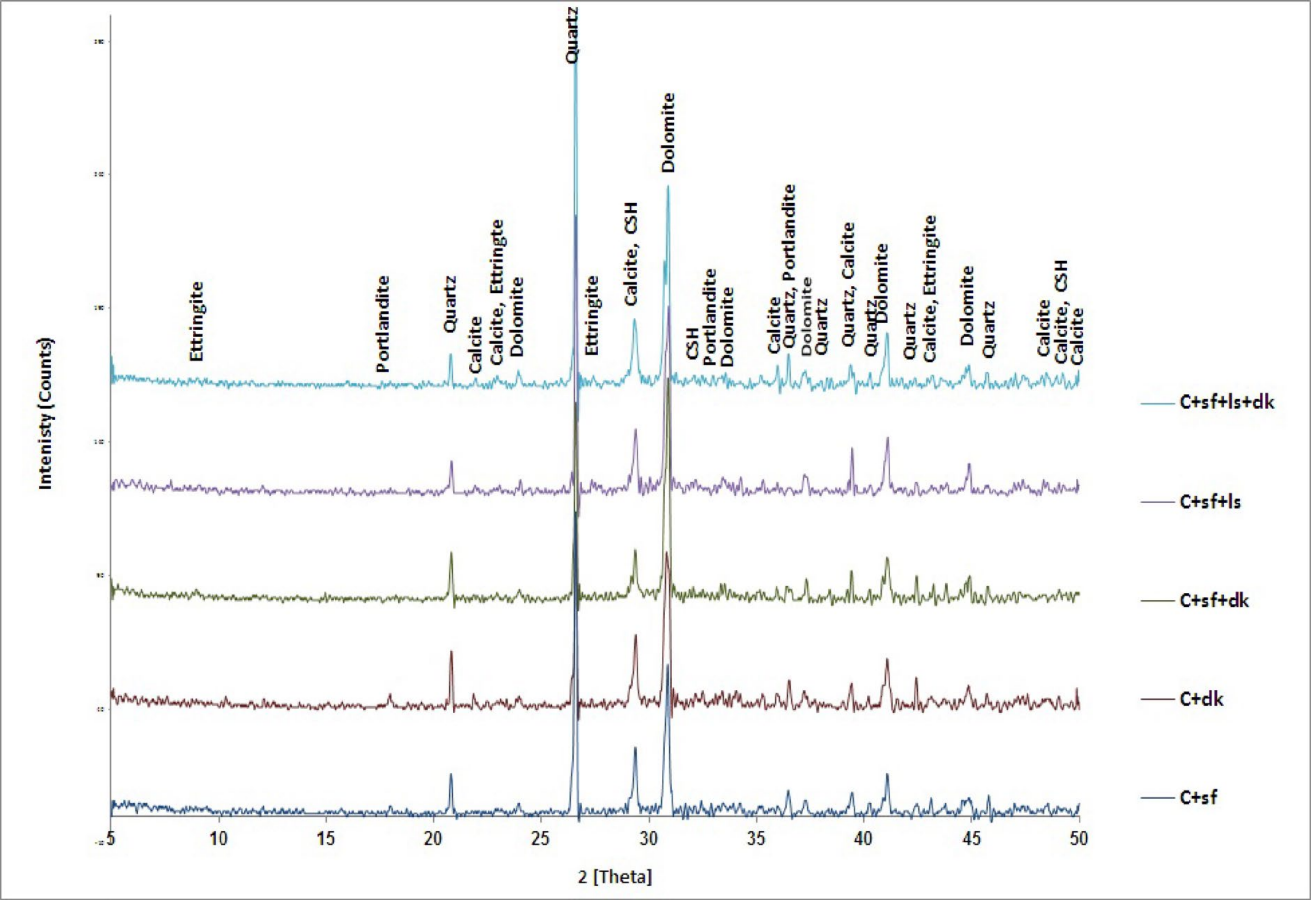
XRD patterns for SF15 and the other cement composites.

X-Ray Diffraction analysis has been performed on four concrete mixes containing the optimum content obtained from strength results at (DK10, SF15LS10, SF15DK10 and SF15LS10DK10) compared to the SF15  mix. The XRD patterns provided crucial insights into the phase development of the concrete mixtures. A pronounced increase in CSH peak intensity was observed for DK10 relative to SF concrete (Fig. 11), signifying an accelerated formation of the primary strength-contributing phase. This was corroborated by a corresponding decrease in portlandite peak intensity, indicative of its consumption via pozzolanic reactions with the high-silicate components of SF15 and DK10. This finding robustly supports the established pozzolanic behavior of SF and DK powders in generating supplementary CSH gel from liberated calcium hydroxide. Moreover, the presence of a more intense unreacted quartz peak in SF15 than in DK10 is directly attributable to the inherent mineralogical composition of silica fume, which possesses higher proportion of quartz. The XRD profile of the SF15LS10 limestone-blended concrete shared many characteristics with SF15, albeit with elevated quartz and sodium alumotrisilicate peaks. A striking disparity, however, was the substantially higher calcite (CaCO_3_) peak intensity in SF15LS10. This can be explained by two principal factors: the direct contribution of calcite from the limestone powder itself, and the potential for partial carbonation of portlandite, a reaction that yields additional calcite. The subtle enhancement in CSH peak intensity within SF15LS10 is plausibly linked to the physical densification and pore-filling effects exerted by the limestone particles, leading to a more compact, homogeneous, and consolidated concrete matrix.

Fig. 11 further presents XRD data that substantiates the enhanced mechanical properties previously reported for cementitious pastes containing pozzolanic powders (SF15LS10DK10, SF15DK10, and SF15LS10). A recurrent observation is the diminished intensity of CH peaks in SF15LS10DK10, SF15LS10, and SF15DK10 when contrasted with SF15. This reduction in CH peak intensity strongly implies the direct involvement of pozzolanic powders in the ongoing hydration process. Pozzolanic materials (SF/DK), typically characterized by their significant silica (SiO_2_) content (largely as quartz), actively consume CH, a product of ordinary Portland cement hydration. This pozzolanic reaction pathway facilitates the transformation of CH, a crystalline and less effective binder, into amorphous CSH gel, which is the primary contributor to concrete’s strength. The preferential formation of CSH is consequentially reflected in the heightened intensity of CSH peaks identified through XRD. The quartz peak intensity, however, revealed notable variations between the pozzolanic concrete formulations. Specifically, individual SF concrete mix (SF15) evinced a considerably higher concentration of unreacted quartz than DK concrete (DK10). This observation prompts the exploration of two distinct mechanistic or compositional factors. Firstly, it could be indicative of a superior pozzolanic reaction efficiency within the DK concrete. An intensified consumption of silica by CH in DK concrete would inherently lead to a lower residual unreacted quartz phase. Secondly, the disparity in quartz peak intensity might be ascribed to fundamental differences in the initial chemical composition of the raw DK and SF powders. Should DK powder possess an intrinsically lower initial silicate content compared to SF15, then a reduced proportion of quartz would naturally be detected in its XRD spectrum, even if both powders exhibited similar pozzolanic reactivities.

#### SEM analysis results

**Fig. 12:**
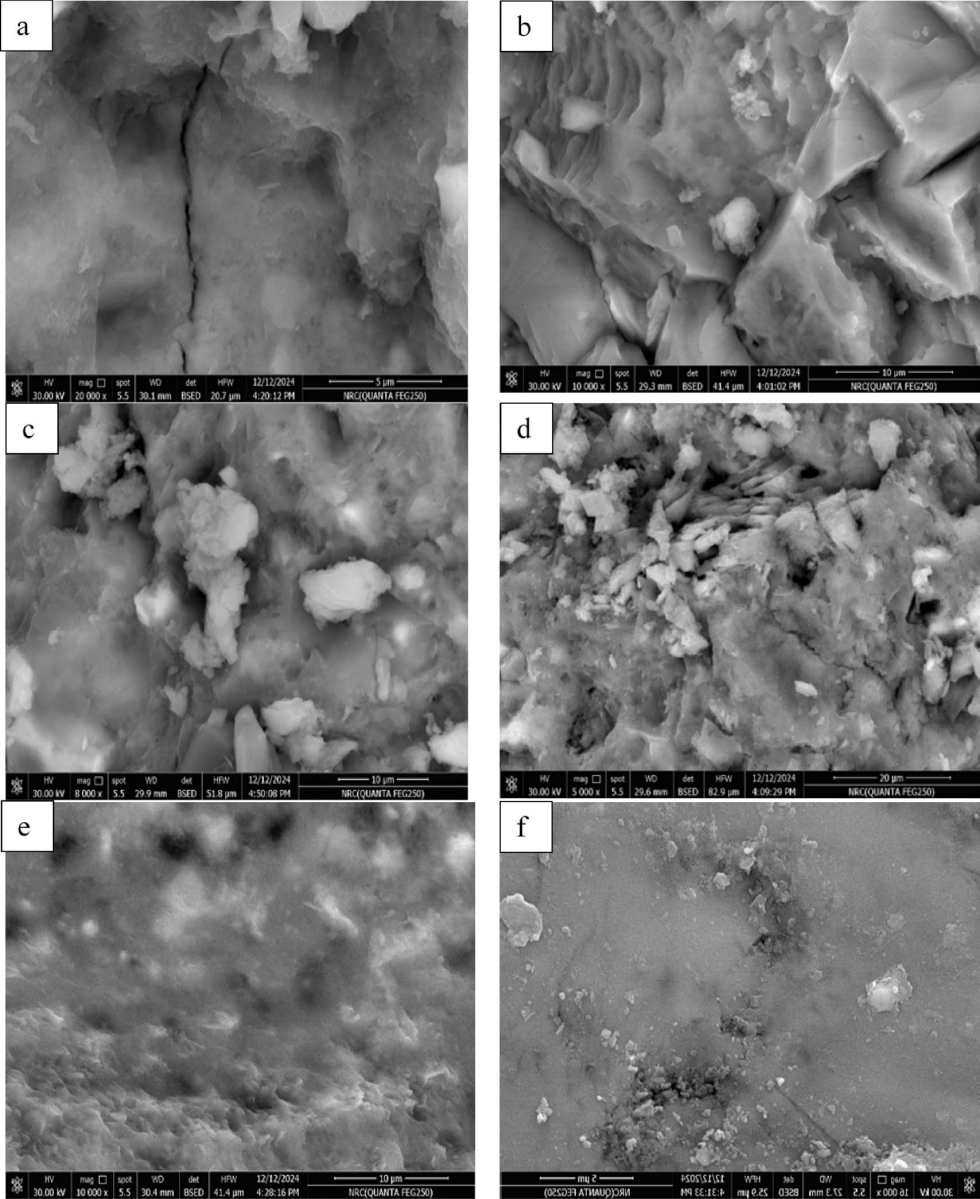
SEM micrographs for (**a**) CO, (**b**) SF 15%, (**c**) DK 10%, (**d**) SF15DK10%, (**e**) SF15LS10% and (**f**) SF15LS10DK10%.

Fig. 12 illustrates SEM analysis that was conducted to investigate the morphology and microstructure of the different concrete mixtures. This resulted in the generation of C–H, C–S–H, ettringite, pores and cracks clear images to understand further the mechanical properties of the microstructures of concrete. Control sample images show a highly porous microstructure with wide pores. It shows large and wide cracks that make the matrix heterogeneous. In addition, SEM images of control samples show the higher concentration of plate-shaped C-H crystals, which is indication of unconsumed CH during the hydration phase, lead to weakness in mechanical properties. In comparison, the micrograph of concrete with replacing of cement by LS 10%, the porosity is minimized due to presence of LS and the filling impact of LS particles in the concrete, which can be seen in SEM images with the same size and shape in its SEM images. Further, sponge-like C–S–H gel is observed with longer needles ettringite in the matrix of specimen containing LS. This is due to the occupation of pores by LS particles and restricts the growth of calcium hydroxide crystals and formation more C–S–H.

The micrograph of concrete with replacement of cement by SF15% and DK10% revealed a dense, more homogenous and more compacted compared to control mix. The micrograph deposits the absence of pores, cracks and C–H crystals. Conversely, it was observed that a large amount of C–S–H was found in comparison with control mix. The presence of plenty of C–S–H gel with the absence of C–H crystals is an indication of a higher degree of hydration due to pozzolanic reaction of SF and DK particles.

The role of SF and DK represented by the continuity of hydration process between C–H and SF and DK particles as a pozzolanic material, producing more C–S–H gel, which has the potential to result in better mechanical properties. The micrograph of concrete with addition SF, DK and LS shows significantly fewer pores and denser microstructure compared to the other concrete samples. Adding SF, DK and LS particles to concrete reduces the porosity value and makes the concrete more compact.

#### EDX results

**Fig. 13:**
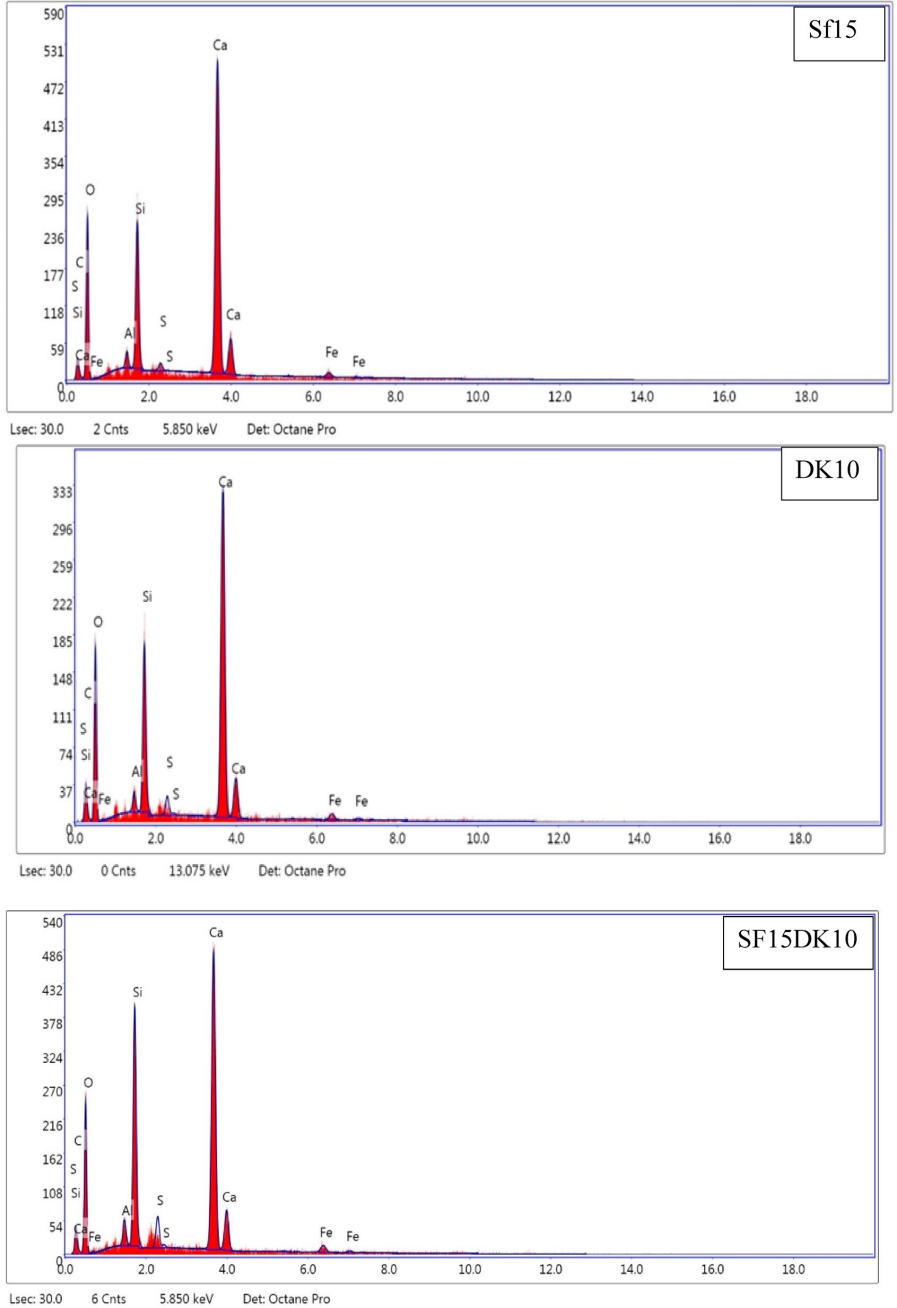

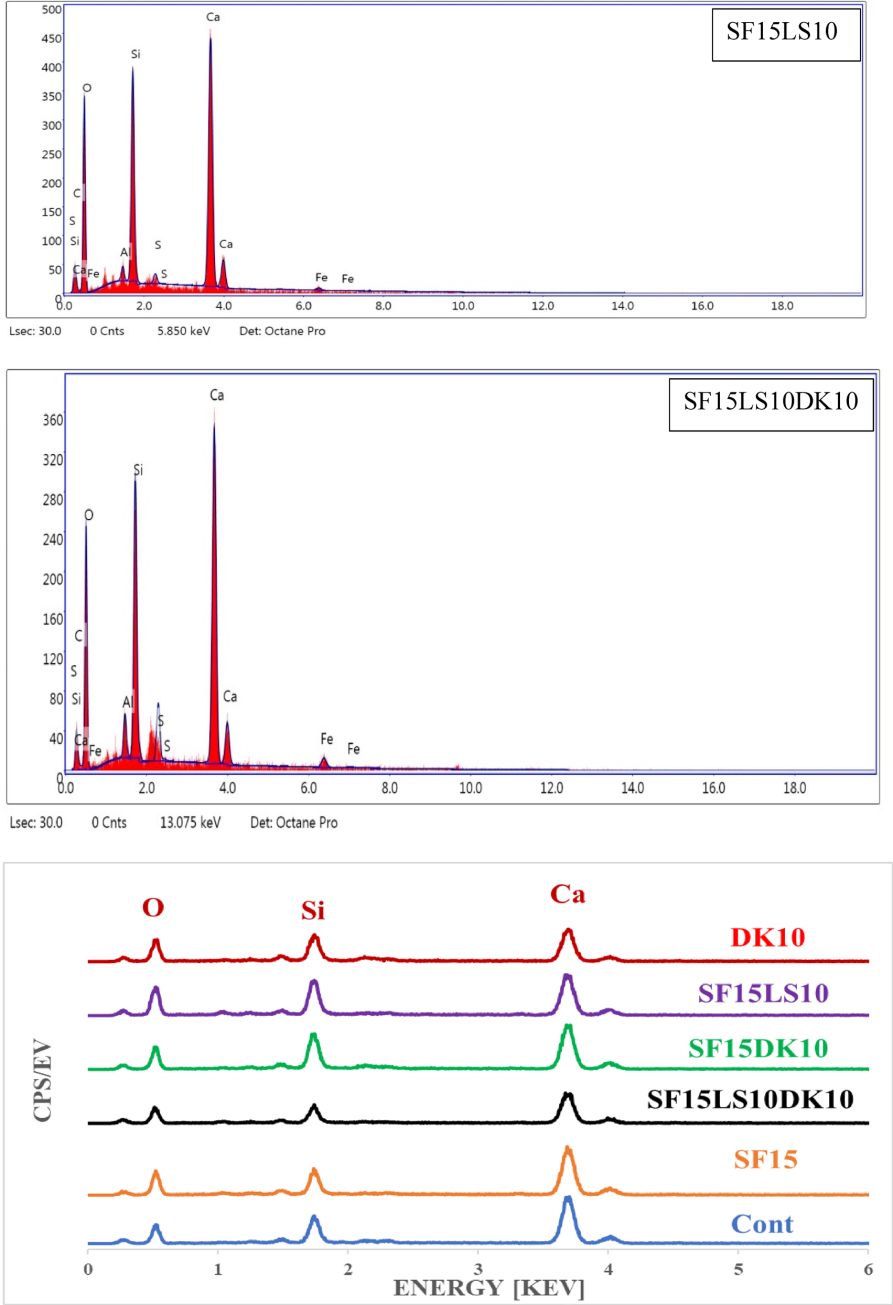
EDX patterns for the control and optimum waste powder additive mixes.

EDX analysis provides the relative weight percentages of constituent elements within the analyzed samples. These data are directly integrated as input parameters into simulation models developed to predict the radiation scattering characteristics of HSC samples containing SF, DK, and LS. Furthermore, EDX uniquely enables the detection of organic compounds by identifying their constituent carbon, addressing a limitation of XRF which cannot detect such compounds under its high-temperature conditions. Table 5 summarizes the EDX findings for five analyzed high-strength concrete mixes. The EDX patterns detailing the major elemental composition (O, Ca, Si, and Al) within the hydration products of various concrete specimens are depicted in Fig. 13 Additionally, Table 6 provides the calculated Ca/Si and Ca/Al ratios, with the Ca/Si ratio serving as a critical metric for assessing C–S–H formation and overall concrete strength. The results show that the Ca/Si ratio equals (4.72, 4.16, 3.85, 2.52, 2.38and 2.37) in case of (CO, SF15, DK10, SF15DK10, SF15LS10 and SF15LS10DK10), respectively.

**Table 5:** EDX results for the various HSC mixes.

Element	Percent by wt.%					
	Co	SF15	DK10	SF15DK10	SF15LS10	SF15LS10DK10
O K	46.01	51.57	48.85	45.65	52.32	50.01
Ca K	36.42	32.81	30.59	29.95	25.99	25.41
Si K	7.72	7.88	7.95	11.89	10.93	10.7
Al K	1.33	1.04	1.22	1.55	1.44	1.88
Fe K	0.68	1.28	1.53	1.87	1.62	1.92
C K	6.87	4.96	8.86	7.2	7.1	7.59
SK	0.95	0.44	0.97	1.88	0.59	2.47
Density (gm/cm^3^)	2.39	2.43	2.47	2.42	2.44	2.45

**Table 6:** Ca/Si ratio and Ca/Al ratio for different concrete mixes.

Sample	CO	SF15	DK10	SF15DK10	SF15LS10	SF15LS10DK10
Ca/Si ratio	4.72	4.16	3.85	2.52	2.38	2.37
Ca/Al ratio	37.38	31.55	25.1	19.32	18.04	13.52

The systematic reduction in the Ca/Si ratio (CO > SF15 > DK10 > SF15DK10 > SF15LS10 > SF15LS10DK10) is a critical indicator of improved concrete performance. This decrease aligns with existing literature^67–70^ that links lower Ca/Si ratios to higher compressive strength through the development of longer and more polymerized C–S–H chains. Concurrently, the Ca/Al ratio also showed a consistent decline across the mixes, from 37.38 for CO to 13.52 for SF15LS10DK10 (Table 6). A lower Ca/Al ratio signifies increased alumina content and reduced portlandite, which is conducive to the formation of calcium aluminate hydrate (C-A-H), a phase known to bolster early-age strength^71^. The particularly low Ca/Al ratios in SF15LS10 and SF15LS10DK10 relative to the control (CO) indicate a preferential reaction of alumina with portlandite to generate C-A-H. The synergistic effect of a decreasing Ca/Si ratio (enhancing C–S–H quality) and a lower Ca/Al ratio (promoting C-A-H formation) collectively accounts for the superior compressive strength achieved in these specific concrete formulations.

### Radiation shielding results of HSC

#### Gamma ray attenuation

**Fig. 14:**
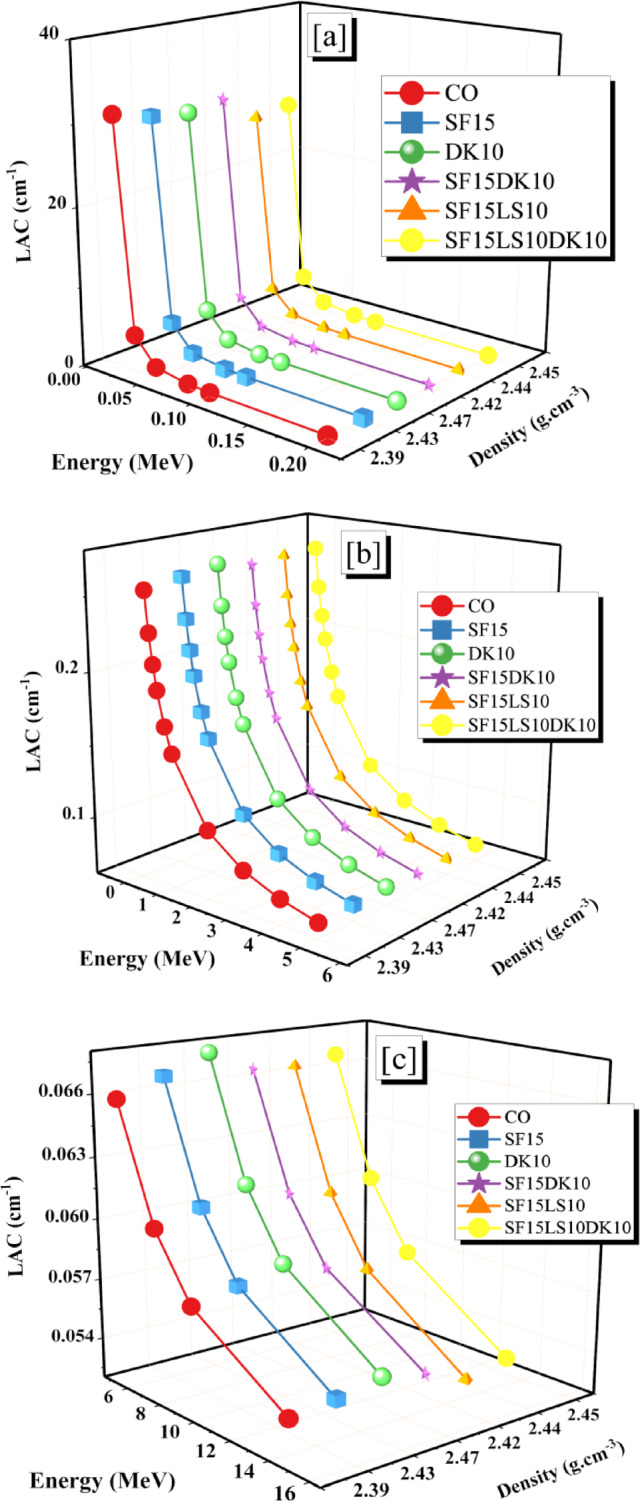
**(a–c):** The linear attenuation (**a**) in the PEE region, (**b** and **c**) in the Compton region for the prepared concrete samples.

The LAC results obtained by MC and Phy-X in the photon energy (γp) range of 0.015–15 MeV were in decent agreement (with a maximum Δ of 4.168%). The LAC value of the concrete samples under investigation decreases as the γ-energy rises. For the concrete samples, the simulated LAC values decline as follows: 31.180 to 0.054 cm^−1^ for CO sample, 29.926 to 0.055 cm^−1^ for SF15 sample, 29.347 to 0.055 cm^−1^ for DK10 sample, 29.989 to 0.055 cm^−1^ for SF15DK1 sample, 26.602 to 0.054 cm^−1^ for SF15LS10 sample, and 27.359 to 0.054 cm^−1^ SF15LS10DK10 sample at γp range from 0.015 to 15 MeV. Fig. 14a shows an abrupt decrease in LAC for each of the concrete samples under study because of the PEE interaction, which has changed the cross-section (σp) with γ ^−3: 5^^72–74^. It’s observed that when the values of γp energy increase from 0.015 to 0.2 MeV, σp decreases significantly, and the PEE interaction consequently decreases. For concrete samples, there is a strong tendency to decrease from 31.180 to 0.302 cm^−1^ for CO sample, 29.926 to 0.306 cm^−1^ for SF15 sample, 29.347 to 0.310 cm^−1^ for DK10 sample, 29.989 to 0.305 cm^−1^ for SF15DK1 sample, 26.602 to 0.306 cm^−1^ for SF15LS10 sample, and 27.359 to 0.308 cm^−1^ SF15LS10DK10 sample.

The expected LAC in the γp range from 0.300 to 15 MeV decreases exponentially when γp from 0.300 to 15 MeV is increased, as shown in Fig. 14b and c. The CM interaction and the σp changes brought on by γp^−1^ are responsible for the exponential decline^65,75,76^. This effect can be explained by the fact that because of its increased speed, a larger γp has a lower tendency to interact with the atoms of the material. Consequently, the probability of γp interaction decreases, and the likelihood of γp scattering increases as the energy level rises.

**Fig. 15:**
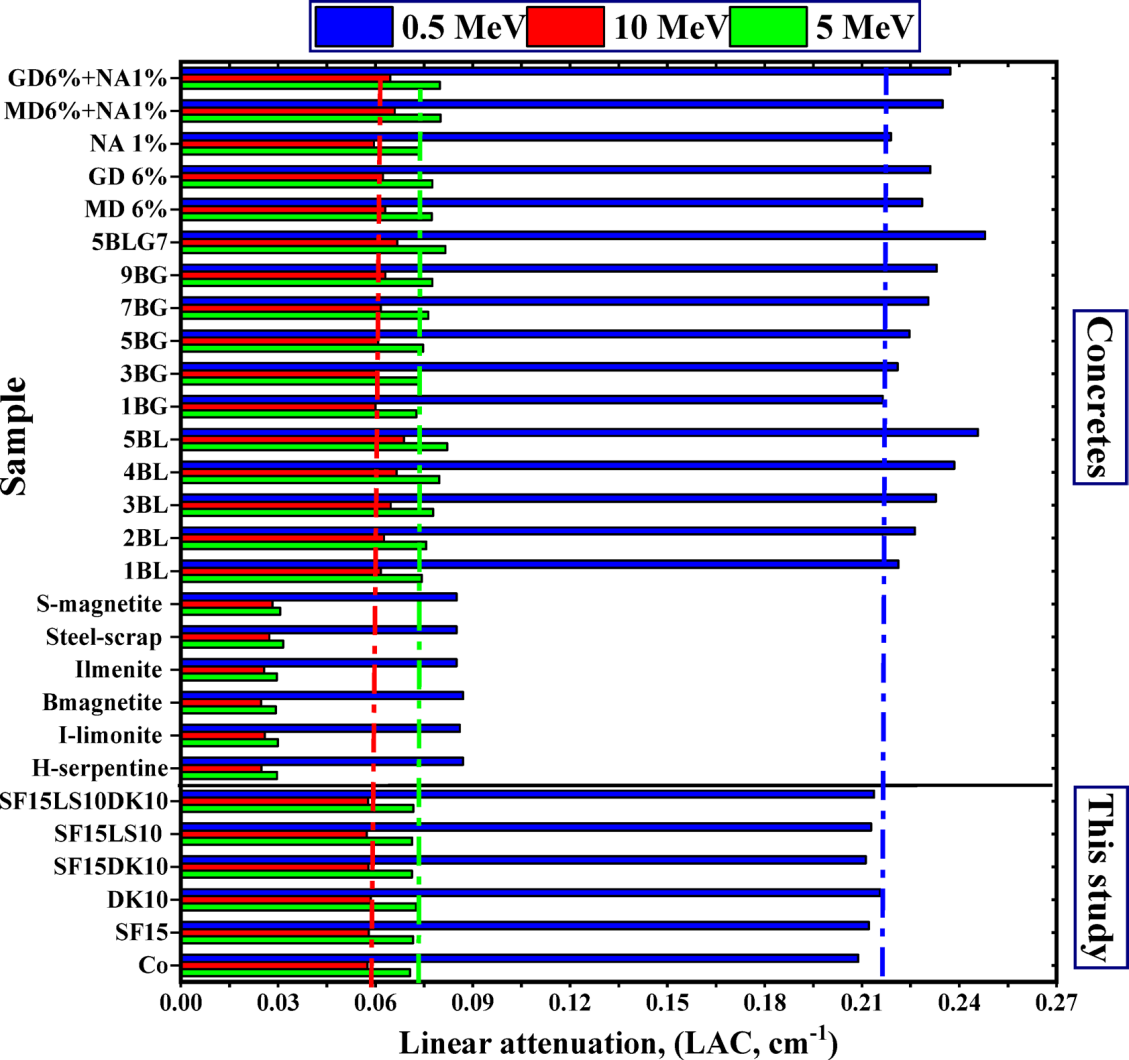
Comparison between the LAC values for HSC samples in this study with different literature results.

A smooth decline in LAC values followed a gradual decrease in σp with fewer electron-γp interactions, which was linked to an increase in γp values. So, the LAC values gradually decrease from 0.257 to 0.054 cm^−1^ for the CO sample, 0.260 to 0.055 cm^−1^ for the SF15 sample, 0.264 to 0.055 cm^−1^ for the DK10 sample, 0.259 to 0.055 cm^−1^ for SF15DK1 sample, 0.261 to 0.054 cm^−1^ for SF15LS10 sample, and 0.262 to 0.054 cm^−1^ for SF15LS10DK10 sample. The highest LAC was observed in the HSC (DK10) and (SF15LS10DK10) samples. This can be directly attributed to their superior physical properties, specifically their high density (2.47 and 2.45 g.cm^−3^) and high Fe element content (1.53 and 1.92%), in that order. The LAC for the mixed concrete samples and previously published doped concrete samples are compared in Fig. 15. As seen in the figure, the sample DK10 investigated in the current study achieved results equal to some of previously studied and published samples at 10 MeV, 0.5 MeV, and 5 MeV. It presents a comparison of the HSC samples with commercial concrete (mixes of PbO bulk powder with cement, with substitution ratios ranging from 1 to 5% %. Additionally, five other mixtures of GD bulk powder with cement increased the substitution rates to 9% and one hybrid mix combining 5% PbO and 7% GD^58^), Bashtar concretes (S-magnetite, S-scrap, ilmenite, H-serpentine, I-limonite, and B-magnetite^77^), and marble (MD) and granite (GD) waste dust of cement at a replacement ratio of 6%. Furthermore, two additional mixes with the cement weight of nano-alumina (NAl)^57^. The prepared concrete mix (DK10) was higher than those compared (Bashtar concretes, and 1BG) at 0.5 and 5 MeV. At 10 MeV The prepared concrete mix (DK10) was higher than those compared (Bashtar concretes, and NA1%).

Three popular parameters are used to measure the efficacy of radiation shielding: the MFP, HVT, and TVT^53,78,79^. This illustrates both the shielding material’s thickness and radiation-blocking capabilities. For a given photon energy, the radiation shielding performance is enhanced by lowering either value. This is because when the value is reduced, radiation is attenuated through a smaller zone^80,81^. The values of HVT and TVT have an inverse relationship with the LAC. As the LAC dropped, the synthesized concrete samples’ HVT rose from 0.022 to 12.743 cm for CO sample, 0.023 to 12.688 cm for SF15 sample, 0.024 to 12.597 cm for DK10 sample, 0.023 to 12.706 cm for SF15DK1 sample, 0.026 to 12.919 cm for SF15LS10 sample, and 0.025 to 12.813 cm for SF15LS10DK10 sample at γp range from 0.015 to 15 MeV.

**Fig. 16:**
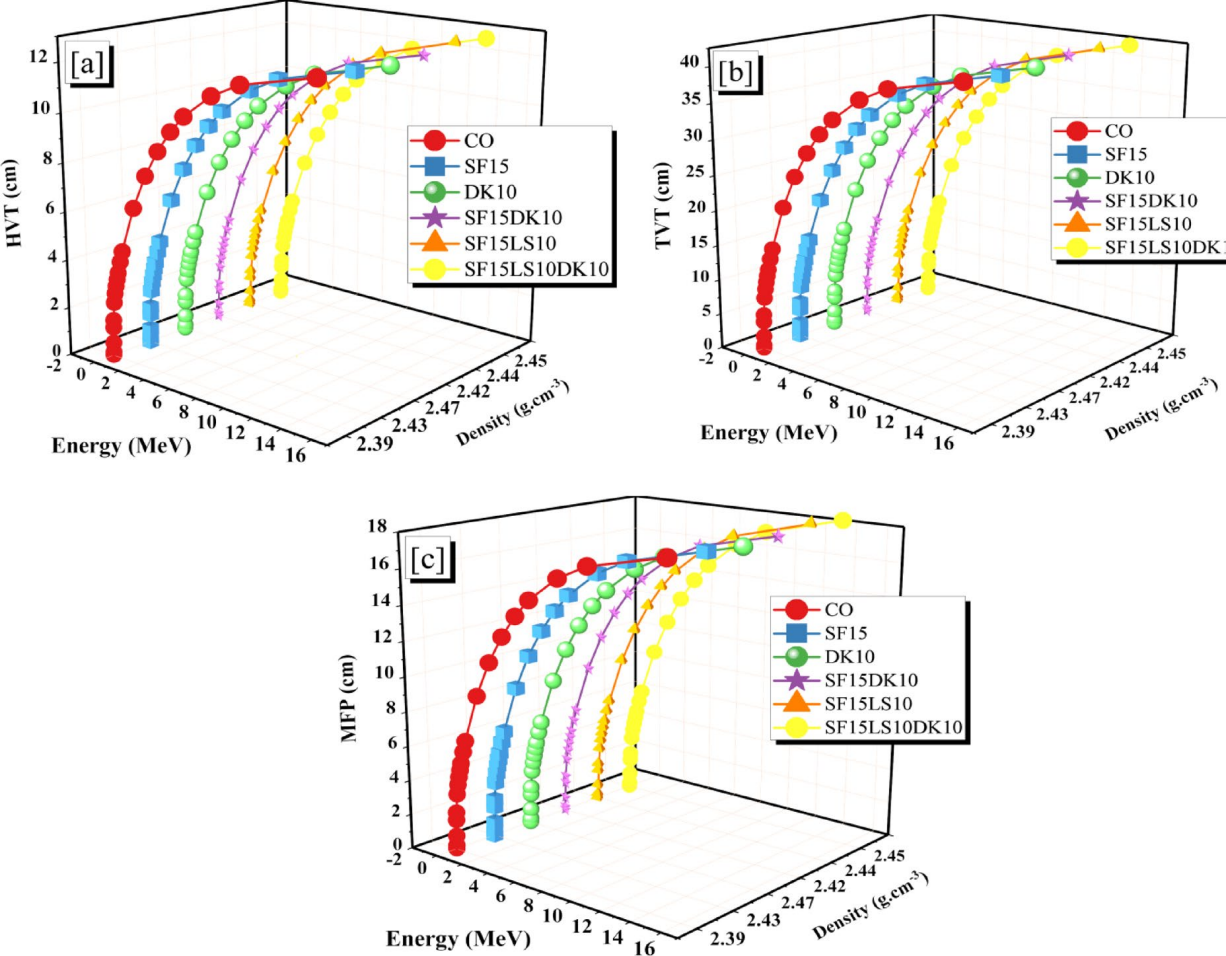
**(a-c)**: displays the concrete samples’ HVT, TVT, and MFP in relation to γ-energy.

The SF15DK1 sample has the lowest HVT value because of its highest LAC value, whereas the SF15LS10DK10 sample has the highest HVT value because of its low LAC value, as shown in Fig. 16a. HVT values show a pattern similar to that of HVT, as shown in Fig. 16b, concrete has the best radiation shielding properties, and the addition of high-density compounds enhances the γ attenuation capabilities within the selected γe range from 0.015 to 15 MeV.

The TVT values increased for the CO sample by 0.074 to 42.332 cm, the SF15 sample by 0.077 to 42.147 cm, the DK10 sample by 0.077 to 42.209 cm, the SF15DK10 sample by 0.087 to 42.917 cm, the SF15LS10 sample by 0.087 to 42.917 cm, and the SF15LS10DK10 sample by 0.084 to 42.563 cm. Because of its high LAC value, the DK10 sample has the lowest TVT. Understanding how well various materials absorb or scatter gamma rays is made easier by studying the MFP. The more effectively a material reduces ray passage through it, the lower the mean free path^82–84^. Therefore, in order to protect people and equipment from the effects of gamma radiation, it is crucial to study the main free path when choosing and designing the materials used as protective shields in nuclear and industrial applications^85^. The MFP values for the concrete samples increase as γp energies increase from 0.015 to 15 MeV taking the same trend. MFP values are lowest in the DK10 and SF15LS10DK10 samples as shown in Fig. 16c and that is due to the inverse relationship between MFP and LAC^86^.

**Fig. 17:**
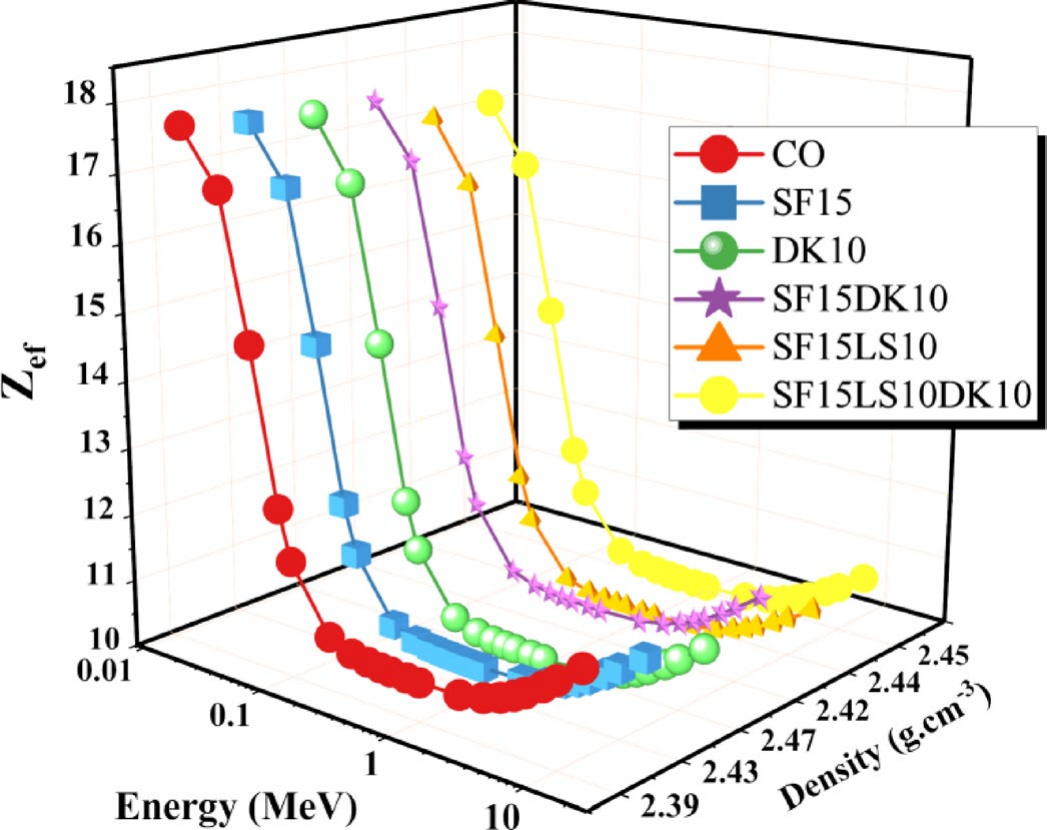
The Z_ef_ for the concrete samples as a function of photon energy.

For the concrete samples, Fig. 17 displays graphs of the adequate atomic number versus γ-energy ranging from 0.015 to 15 MeV. A higher Z_ef_ value indicates a greater interaction with radiation through mechanisms like PEE/Compton interactions. Materials with a higher Z_ef_ value may be better for protecting against high-energy^87^. As the γp rises for the materials under study, the Z_ef_ values fall. The concrete’s Z_ef_ ranged from 17.666 to 11.557 for CO, 17.530 to 11.291 for SF15, 17.456 to 11.013 for DK10, 17.473 to 11.378 for SF15DK1, 17.020 to 10.797 for SF15LS10, and 17.095 to 10.907 for SF15LS10DK10 concrete sample, depending on the energy spectra specified in this study. Given this, it is feasible to conclude that the radiation shielding efficiency of materials varies with the radiation energy; that is, certain substances may be more effective at higher or lower energies than others^88^. Due to its content of elements with a high atomic number and its high density compared to the other samples in this study, the DK10 sample had the highest value of Z_ef_.

#### Neutron attenuation

**Fig. 18:**
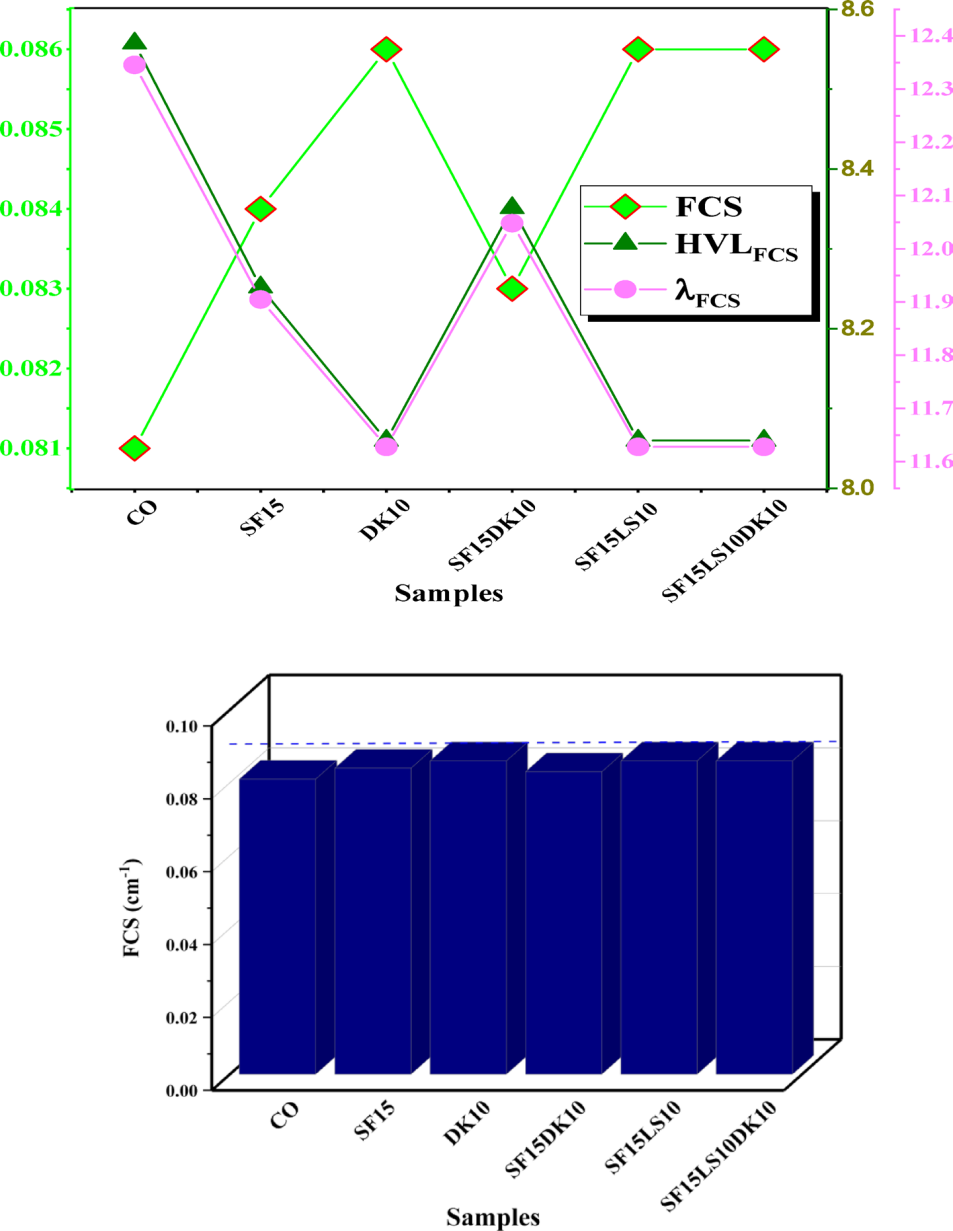
**(a)** The mixed concrete’s fast neutron removal cross-section (FCS), half value layer (HVL_FCS_), and relaxation length (λ_FCS_). (**b)** The fast neutron removal cross-section (FCS) for the prepared samples.

In this study, the fast neutron removal cross-section (FCS), half value layer (HVL)_FCS_, and the relaxation length (λ)_FCS_values for the HSC samples are displayed in Fig. 18a and b. In contrast to the other samples in this study, the DK10, SF15LS10, and SF15LS10DK10 samples had the same highest value for FCS (0.086 cm^−1^) and the lowest values for HVL_FCS_ (8.059 cm) and λ_FCS_ (11.627 cm), indicating its efficacy and capability as a neutron shield. The high density and concentration of light elements (carbon and oxygen) was the cause^57,58,77^.

## Conclusions

This study investigated the influence of incorporating waste blended powders of silica fume (SF), limestone (LS), and dealuminated metakaolin (DK) on high-strength concrete (HSC) properties. The experimental study involved designing nine different HSC mixes incorporating binary, ternary, and quaternary cement replacements at varying substitution levels: SF15, DK5, DK10, SF15DK5, SF15DK10, SF15LS10, SF15LS10DK5, and SF15LS10DK10. To thoroughly characterize these materials, the properties of the primary concrete constituents, such as cement, aggregates, and additives, were examined. The produced concrete samples were then subjected to a wide array of tests. These tests included the slump cone to evaluate workability, compressive and tensile strength tests for mechanical properties assessment, and microstructural analyses using X-ray Diffraction (XRD), Scanning Electron Microscopy (SEM), and Energy-Dispersive X-ray (EDX) . Significantly, EDX data and measured densities were instrumental in developing simulation models, executed via Monte Carlo (MC) methods and Phy-X software, to evaluate the radiation shielding efficiency for gamma (γ)-rays and fast neutrons through the diverse investigated HSC mixes. The main conclusions derived from this comprehensive experimental program and simulation results can be summarized as the following:

1. Incorporating SF, LS, and DK waste blended powders into HSC as a partial cement replacement material consistently reduced its workability, as evidenced by a consistent decrease in slump values. This effect is largely attributable to the high fineness of the used SCMs which increased water demand, the development of a more compact concrete microstructure, and elevated internal friction. Furthermore, the synergistic effects observed in the ternary, and quaternary blends led to more significant slump reduction than when these materials were incorporated individually.
2. The incorporation of SF, LS, and DK significantly enhanced the compressive strength of concrete. Among binary blends, 10% DK achieved the highest compressive strength improvement (37.2%), compared to 17.7% with the 15% SF mix. Ternary blends of 15% SF with 5% DK, 10% DK, or 10% LS improved compressive strength by 39.1%, 23.4%, and 29.15%, respectively. Quaternary blends of 15% SF + 10% LS with 5% or 10% DK yielded increases of 28.3% and 32.4%. These consistent strength gains stem from the synergistic pozzolanic activity of the components, which promotes reduced porosity and stronger interfacial bonding.
3. Binary, ternary, and quaternary blends using SF, LS, and DK significantly improved tensile strength. The optimal ratios, recorded in DK10, SF15DK10, and SF15LS10DK10 mixes, showed improvements of 43.35%, 15.3%, and 22.25%, respectively, compared to the control mix.
4. XRD analysis confirmed increased CSH formation with SF, LS, and DK, correlating with the improved mechanical properties. This was attributed to pozzolanic reactions between SF/DK and portlandite (CH), evidenced by the reduced CH peak intensity. The presence of SF and DK also led to higher quartz and kaolinite peaks, reflecting their silica- and kaolinite-rich compositions.
5. EDX analysis provided insights into elemental composition, revealing that the incorporation of SF, LS, and DK resulted in a reduction of the Ca/Si ratio. This reduction is typically associated with increased C–S–H formation, a key factor in boosting compressive strength. Notably, the SF15LS10DK10 quaternary blend also exhibited a decreased Ca/Al ratio, signifying enhanced C-A-H formation, which further contributes to the overall strength development.
6. γ-ray attenuation tests demonstrated a modest improvement in the γ-ray shielding capacity of the investigated HSC. The linear attenuation of the prepared sample which contains SF15LS10DK10 has the highest LAC, respectively because of its high density (2.47, 2.45 g cm^−3^) as well as the high content of the Fe element (1.53, 1.92%).
7. The samples DK10, SF15LS10, and SF15LS10DK10 had the highest value for FCS (0.086 cm^−1^) and the lowest values for HVL_FCS_ (8.059 cm) and λ_FCS_ (11.627 cm), indicating its efficacy and capability as a neutron shield.

Finally, while this study offers important insights into the mechanical, microstructural, and radiation shielding properties of sustainable high-strength concrete incorporating silica fume, limestone, and dealuminated metakaolin, some limitations should be acknowledged. The study considered only specific replacement levels of these materials, which restricts the understanding of their full potential and the possibility of achieving more optimized blends. In addition, the evaluation focused primarily on short-term mechanical properties and microstructure, whereas the long-term behavior under aggressive environmental conditions such as high temperature exposure, chemical attack, freeze-thaw cycles, and combined loading scenarios requires further exploration. Future research should therefore address these aspects and extend the investigation to large-scale structural applications in order to validate the practical feasibility of the proposed concrete mixes.

## Data Availability

All data generated or analyzed during this study are included in this published article.

## References

[1] Shah, A. A. & Ribakov, Y. Recent trends in steel fibered high-strength concrete. *Mater. Des.* **32**(8–9), 4122–4151 (2011).

[2] Choe, G. et al. Evaluation of the mechanical properties of 200 MPa ultra-high-strength concrete at elevated temperatures and residual strength of column. *Constr. Build. Mater.* **86**, 159–168 (2015).

[3] Behnood, A. & Ziari, H. Effects of silica fume addition and water to cement ratio on the properties of high-strength concrete after exposure to high temperatures. *Cement Concr. Compos.* **30**(2), 106–112 (2008).

[4] Heikal, M. et al. Behavior of composite cement pastes containing microsilica and fly ash at elevated temperature. *Constr. Build. Mater.* **38**, 1180–1190 (2013).

[5] Fathy, I.N. et al. Enhancing mechanical and radiation shielding properties of concrete with lead monoxide and granodiorite: Individual and synergistic effects at micro and nano particle scales. *Structural Concrete.* **26**(2), 1180–1190 (2025).

[6] El-Mir, A. & El-Zahab, S. Assessment of the compressive strength of self-consolidating concrete subjected to freeze-thaw cycles using ultrasonic pulse velocity method. *Russ. J. Nondestr. Test.* **58**(2), 108–117 (2022).

[7] Shah, S., Akashah, F. & Shafigh, P. Performance of high strength concrete subjected to elevated temperatures: A review. *Fire Technol.* **55**, 1571–1597 (2019).

[8] Ahmad, F. et al. Fire resistance and thermal performance of hybrid fibre-reinforced magnesium oxychloride cement-based composites. *Constr. Build. Mater.* **472**, 140867 (2025).

[9] El Mir, A. & Nehme, S. G. Effect of air entraining admixture on the properties of self-compacting concrete incorporating supplementary cementitious materials. *Pollack Periodica* **12**(3), 85–98 (2017).

[10] Amran, M. et al. Global carbon recoverability experiences from the cement industry. *Case Stud. Constr. Mater.* **17**, e01439 (2022).

[11] Mishra, U. C., Sarsaiya, S. & Gupta, A. A systematic review on the impact of cement industries on the natural environment. *Environ. Sci. Pollut. Res.* **29**(13), 18440–18451 (2022). 10.1007/s11356-022-18672-7 35037150

[12] Refaat, M. et al. Minimizing energy consumption to produce safe one-part alkali-activated materials. *J. Clean. Prod.* **323**, 129137 (2021).

[13] El-Feky, M. et al. Microstructural investigation for micro-nano-silica engineered magnesium oxychloride cement. *Constr. Build. Mater.* **342**, 127976 (2022).

[14] Nasir, A., Butt, F. & Ahmad, F. Enhanced mechanical and axial resilience of recycled plastic aggregate concrete reinforced with silica fume and fibers. *Innov. Infrastruct. Solut.* **10**(1), 4 (2025).

[15] Kondraivendhan, B. & Bhattacharjee, B. Flow behavior and strength for fly ash blended cement paste and mortar. *Int. J. Sustain. Built Environ.* **4**(2), 270–277 (2015).

[16] Cheah, C. B. et al. Properties of ternary blended cement containing ground granulated blast furnace slag and ground coal bottom ash. *Constr. Build. Mater.* **315**, 125249 (2022).

[17] Mahmoud, A. A. et al. Investigating the effects of granite, marble, granodiorite, and ceramic waste powders on the physical, mechanical, and radiation shielding performance of sustainable concrete. *Ann. Nucl. Energy* **216**, 111274 (2025).

[18] Mostafa, S. A. et al. Optimization of UHPC with basil plant ash: Impacts on strength, durability, and gamma-ray attenuation. *Ann. Nucl. Energy* **226**, 111825 (2026).

[19] Joshaghani, A. & Moeini, M. A. Evaluating the effects of sugarcane-bagasse ash and rice-husk ash on the mechanical and durability properties of mortar. *J. Mater. Civ. Eng.* **30**(7), 04018144 (2018).

[20] Mahmoud, A.A., et al. Synergizing machine learning and experimental analysis to predict post‐heating compressive strength in waste concrete*.**Struct. Concr.* (2025).

[21] Suraneni, P. & Weiss, J. Examining the pozzolanicity of supplementary cementitious materials using isothermal calorimetry and thermogravimetric analysis. *Cem. Concr. Compos.* **83**, 273–278 (2017).

[22] Toutanji, H. et al. Effect of supplementary cementitious materials on the compressive strength and durability of short-term cured concrete. *Cem. Concr. Res.* **34**(2), 311–319 (2004).

[23] Sarıdemir, M. Effect of silica fume and ground pumice on compressive strength and modulus of elasticity of high strength concrete. *Constr. Build. Mater.* **49**, 484–489 (2013).

[24] Xiong, X. et al. Performance and microstructure of ultra-high-performance concrete (UHPC) with silica fume replaced by inert mineral powders. *Constr. Build. Mater.* **327**, 126996 (2022).

[25] Hamdan, F. S., El-Ashgar, N. M. & Musalam, A. M. Respiratory health risks of limestone factories’ dust: Gaza City as a case study. *Israa Univ. J. Appl. Sci.* **5**(1), 74–86 (2021).

[26] Wang, D. et al. A review on effects of limestone powder on the properties of concrete. *Constr. Build. Mater.* **192**, 153–166 (2018).

[27] Ye, G. et al. Influence of limestone powder used as filler in SCC on hydration and microstructure of cement pastes. *Cement Concr. Compos.* **29**(2), 94–102 (2007).

[28] Liu, S. & Yan, P. Effect of limestone powder on microstructure of concrete. *J. Wuhan Univ. Technol.-Mater. Sci. Ed.* 25(2), 328–331 (2010)

[29] Felekoglu, B. Utilisation of high volumes of limestone quarry wastes in concrete industry (self-compacting concrete case). *Resour. Conserv. Recycl.* **51**(4), 770–791 (2007).

[30] Sua-Iam, G. & Makul, N. Use of increasing amounts of bagasse ash waste to produce self-compacting concrete by adding limestone powder waste. *J. Clean. Prod.* **57**, 308–319 (2013).

[31] Ahmad, F. et al. E-waste in concrete construction: Recycling, applications, and impact on mechanical, durability, and thermal properties—A review. *Innov. Infrastruct. Solut.* **10**(6), 246 (2025).

[32] Ahmad, Z. et al. Effect of macro synthetic fiber (MSF) on the behavior of conventional concrete and the concrete containing e-waste aggregates. *Mater. Struct.* **58**(7), 234 (2025).

[33] Fattouh, M. S. et al. Impact of modified aggregate gradation on the workability, mechanical, microstructural and radiation shielding properties of recycled aggregate concrete. *Sci. Rep.* **15**(1), 1–23 (2025). 40419602 10.1038/s41598-025-02655-y PMC12106813

[34] Mahmoud, A. A. et al. Elevated temperature effects on the compressive strength and radiation shielding capability of waste granite and marble concrete. *Eur. Phys. J. Plus* **140**(4), 302 (2025).

[35] Abdulkader, E.-M., Salem, N. & Joseph, A. Feasibility of concrete mixtures containing coarse and/or fine recycled brick aggregates. *Mag. Civil Eng.* **116**(8), 11603 (2022).

[36] Muisa, N. et al. Utilization of alum sludge as adsorbent for phosphorus removal in municipal wastewater: A review. *J. Water Process Eng.* **35**, 101187 (2020).

[37] Abdelalim, A., et al. Dealuminated kaolin as a cement replacement material. *J. Cement Wapno Beton*, 3rd issue (2010).

[38] Ahmad, F. et al. Effect of metakaolin and ground granulated blast furnace slag on the performance of hybrid fibre-reinforced magnesium oxychloride cement-based composites. *Int. J. Civil Eng.* **23**(5), 853–868 (2025).

[39] Mostafa, N. et al. Characterization and evaluation of the pozzolanic activity of Egyptian industrial by-products: I: Silica fume and dealuminated kaolin. *Cem. Concr. Res.* **31**(3), 467–474 (2001).

[40] Abo-El-Enein, S. et al. Reactivity of dealuminated kaolin and burnt kaolin using cement kiln dust or hydrated lime as activators. *Constr. Build. Mater.* **47**, 1451–1460 (2013).

[41] Moselhy, H. Effect of dealuminated kaolin waste on slump and compressive strength for ordinary Portland cement concrete. *Int. J. Chem. Eng.* **10**(2) (2018).

[42] Apte, K. & Bhide, S. Basics of radiation. In *Advanced radiation shielding materials* 1–23 (Elsevier, 2024).

[43] Awadeen, M. et al. Mechanical properties, attenuation coefficient, and microstructure of ultra high-performance heavyweight concrete for radiation shielding applications. *J. Build. Eng.* **82**, 108395 (2024).

[44] Onaizi, A.M., et al. Radiation-shielding concrete: A review of materials, performance, and the impact of radiation on concrete properties. *J. Build. Eng.* 110800 (2024).

[45] Barbhuiya, S., et al. A comprehensive review of radiation shielding concrete: Properties, design, evaluation, and applications. *Struct. Concr.* (2024).

[46] Milasi, S. E., Mostofinejad, D. & Bahmani, H. Improving the resistance of ultra-high-performance concrete against nuclear radiation: Replacing cement with barite, hematite, and lead powder. *Develop. Built Environ.* **15**, 100190 (2023).

[47] ASTM C 150. Standard Specification for Portland cement. American society for testing and materials, West Conshohocken. 2007, PA (USA) (2007).

[48] ASTM International. ASTM C1240-15: Standard specification for silica fume used in cementitious mixtures. West Conshohocken, PA: ASTM International (2015).

[49] ASTM International. (2003). ASTM C33-03: Standard specification for concrete aggregates. West Conshohocken, PA: ASTM International (2003).

[50] ASTM C 1602: standard specification for mixing water used in the production of hydraulic cement concrete (2006).

[51] STM-International, Standard Specification for Chemical Admixtures for Concrete (ASTM C494/C494M-19). ASTM International: West Conshohocken, PA (2019).

[52] Bentz, D. P., Jones, S. Z. & Snyder, K. A. Design and performance of ternary blend high-volume fly ash concretes of moderate slump. *Constr. Build. Mater.* **84**, 409–415 (2015).

[53] El-Samrah, M. G. et al. Microstructure and radiation shielding capabilities of Al–Cu and Al–Mn alloys. *Sci. Rep.* **14**(1), 26721 (2024). 39496684 10.1038/s41598-024-76177-4 PMC11535538

[54] Singh, V. et al. Determination of mass attenuation coefficient for some polymers using Monte Carlo simulation. *Vacuum* **119**, 284–288 (2015).

[55] Al-Ghamdi, H., et al. Investigation of gamma-ray and neutron protection competence of oxyfluoride aluminosilicate glasses reinforced with TbF3: Comparative study. *Radiat. Phys. Chem*. 112105 (2024).

[56] Şakar, E. et al. Phy-X/PSD: Development of a user friendly online software for calculation of parameters relevant to radiation shielding and dosimetry. *Radiat. Phys. Chem.* **166**, 108496 (2020).

[57] Mahmoud, A. A. et al. Influence of sustainable waste granite, marble and nano-alumina additives on ordinary concretes: a physical, structural, and radiological study. *Sci. Rep.* **14**(1), 22011 (2024). 39317712 10.1038/s41598-024-72222-4 PMC11422509

[58] Fathy, I. N. et al. Enhancing mechanical properties and radiation shielding of high-strength concrete with bulk lead oxide and granodiorite. *Nucl. Eng. Des.* **429**, 113626 (2024).

[59] Fathy, I. N. et al. Upgrading the compressive strength and radiation shielding properties of high strength concrete supported with nano additives of lead monoxide and granodiorite. *Prog. Nucl. Energy* **180**, 105562 (2025).

[60] Nabil, I. M. et al. Superiority of clay composite materials of bentonite intercalated with the bimetallic MOFs-Pb/Cu, and nano magnetite to enhance the gamma and neutron radiation shielding. *Ann. Nucl. Energy* **225**, 111762 (2026).

[61] Akman, F. et al. Assessment of neutron and gamma-ray shielding characteristics in ternary composites: experimental analysis and Monte Carlo simulations. *Radiat. Phys. Chem.* **219**, 111682 (2024).

[62] Mokhtari Dorostkar, M. & Abdi Saray, A. Comparative analysis of Monte Carlo simulations and experimental evaluation of PMMA reinforced with hgo for gamma radiation shielding. *Sci. Rep.* **15**(1), 28286 (2025). 40753342 10.1038/s41598-025-14223-5 PMC12317998

[63] Özdoğan, H. et al. Detailed analysis of gamma-shielding characteristics of ternary composites using experimental, theoretical and Monte Carlo simulation methods. *Polymers* **16**(13), 1778 (2024). 39000635 10.3390/polym16131778 PMC11244092

[64] Allam, E. A. et al. New ternary composite to enhance the radiation shielding based on attapulgite clay mixed with bimetallic nano MOFs and nano hematite: Experimental and simulation study. *Constr. Build. Mater.* **463**, 140009 (2025).

[65] Yousef, H. A. et al. Nuclear radiation attenuation properties of PbO-doped polymeric composites. *J. Mater. Sci.: Mater. Electron.* **36**(8), 457 (2025).

[66] Ardalan, R. B., Joshaghani, A. & Hooton, R. D. Workability retention and compressive strength of self-compacting concrete incorporating pumice powder and silica fume. *Constr. Build. Mater.* **134**, 116–122 (2017).

[67] Kim, J. J., Foley, E. M. & Taha, M. M. R. Nano-mechanical characterization of synthetic calcium–silicate–hydrate (C–S–H) with varying CaO/SiO_2_ mixture ratios. *Cement Concr. Compos.* **36**, 65–70 (2013).

[68] Fathy, I. N., Elfakharany, M. E. & El-Sayed, A. A. Recycling of waste granodiorite powder as a partial cement replacement material in ordinary concrete. *Adv. Mater. Sci.* **24**(3), 56–88 (2024).

[69] Dadsetan, S. & Bai, J. Mechanical and microstructural properties of self-compacting concrete blended with metakaolin, ground granulated blast-furnace slag and fly ash. *Constr. Build. Mater.* **146**, 658–667 (2017).

[70] Zhan, B. J., Xuan, D. X. & Poon, C. S. The effect of nanoalumina on early hydration and mechanical properties of cement pastes. *Constr. Build. Mater.* **202**, 169–176 (2019).

[71] Abouelnour, M. A. et al. Recycling of marble and granite waste in concrete by incorporating nano alumina. *Constr. Build. Mater.* **411**, 134456 (2024).

[72] El-Samrah, M. et al. Radiation shielding properties of modified concrete mixes and their suitability in dry storage cask. *Prog. Nucl. Energy* **148**, 104195 (2022).

[73] Kassem, S. M. et al. Optical and radiation shielding properties of PVC/BiVO_4_ nanocomposite. *Sci. Rep.* **13**(1), 10964 (2023). 37415084 10.1038/s41598-023-37692-y PMC10326021

[74] Saleh, A., et al. The role of MoO_3_ on the physical, elasto-mechanical and nuclear shielding efficiency of barium-boro-bismuthate glass system: Comparative investigation. *Mater. Chem. Phys.* 129574 (2024).

[75] Ekinci, N. et al. Impacts of the colemanite on the enhancement of the radiation shielding capacity of polypropylene. *J. Mater. Sci.: Mater. Electron.* **33**(25), 20046–20055 (2022).

[76] Elsafi, M. et al. Unveiling the radiation shielding efficacy of diorite, granodiorite, tonalite, and granite: experimental and simulation study. *Sci. Rep.* **15**(1), 804 (2025). 39755723 10.1038/s41598-024-82081-8 PMC11700117

[77] Al-Ghamdi, H. et al. Investigation of gamma-ray and neutron protection competence of oxyfluoride aluminosilicate glasses reinforced with TbF3: Comparative study. *Radiat. Phys. Chem.* **224**, 112105 (2024).

[78] Mahmoud, K. et al. Optimising shielding capacity: Pressure effects on Diatomaceous Earth Composite materials mixed with sodium silicate. *SILICON* **16**(16), 5921–5932 (2024).

[79] El-Seidy, A. M. A. et al. Preparation, physical, optical, ESR and gamma-ray attenuation efficacy investigation of copper oxide/silver borosilicate glass. *Sci. Rep.* **14**(1), 25354 (2024). 39455675 10.1038/s41598-024-75017-9 PMC11512057

[80] Negm, H. H. et al. Exploring the potential of attapulgite clay composites containing intercalated nano-cadmium oxide and nano-nickel oxide for efficient radiation shielding applications. *Radiat. Phys. Chem.* **225**, 112149 (2024).

[81] Al-Saleh, W. M. et al. Gamma-ray shielding investigation of nano-and microstructures of SnO on polyester resin composites: Experimental and theoretical study. *E-Polymers* **24**(1), 20240039 (2024).

[82] Al-Saleh, W. M. et al. A comprehensive study of the shielding ability from ionizing radiation of different mortars using iron filings and bismuth oxide. *Sci. Rep.* **14**(1), 10014 (2024). 38693293 10.1038/s41598-024-60188-2 PMC11063177

[83] Alharshan, G. A. et al. Effect of lanthanum oxide on the radiation-shielding, dielectric, and physical properties of lithium zinc phosphate glasses. *Radiat. Phys. Chem.* **224**, 112053 (2024).

[84] Gunha, J. V. et al. High thermal stability molybdenum-boosted lithium tellurite glass for radiation shielding. *Ceram. Int.* **50**(20), 39046–39058 (2024).

[85] Kaky, K. M. et al. Theoretical and experimental validation gamma shielding properties of B_2_O_3_–ZnO–MgO–Bi_2_O_3_ glass system. *Mater. Chem. Phys.* **242**, 122504 (2020).

[86] Japari, S. et al. Effects of Na_2_O on optical and radiation shielding properties of xNa_2_O-(20_–x_) K_2_O–30V_2_O_5_-50TeO_2_ mixed alkali glasses. *Results Phys.* **22**, 103946 (2021).

[87] Kiran, K. et al. Effective atomic number of selected construction materials using gamma backscattering technique. *Ann. Nucl. Energy* **85**, 1077–1084 (2015).

[88] Almousa, N. et al. Enhancing radiation shielding with gadolinium (III) oxide in cerium (III) fluoride-doped silica borate glass. *Sci. Technol. Nucl. Install.* **2024**(1), 8910531 (2024).

